# Identification and characterization of *Glycolate oxidase* gene family in garden lettuce (*Lactuca sativa cv. ‘Salinas’*) and its response under various biotic, abiotic, and developmental stresses

**DOI:** 10.1038/s41598-023-47180-y

**Published:** 2023-11-11

**Authors:** Mariyam, Muhammad Shafiq, Saleha Sadiq, Qurban Ali, Muhammad Saleem Haider, Umer Habib, Daoud Ali, Muhammad Adnan Shahid

**Affiliations:** 1 Department of Horticulture, University of the Punjab, Lahore, Pakistan; 2 Department of Biotechnology, The Islamia University of Bahawalpur, Bahawalpur, Pakistan; 3 Department of Plant Breeding and Genetics, University of the Punjab, Lahore, 54590 Pakistan; 4 Department of Plant Pathology, University of the Punjab, Lahore, Pakistan; 5 Department of Horticulture, PMAS Arid Agriculture University, Murree Road, Rawalpindi, Pakistan; 6 Department of Zoology, College of Science, King Saud University, PO Box 2455, 11451 Riyadh, Saudi Arabia; 7 Horticultural Sciences Department, North Florida Research and Education Center, University of Florida/IFAS, Quincy, FL 32351 USA

**Keywords:** Biotechnology, Plant biotechnology

## Abstract

Glycolate oxidase (*GLO*) is an FMN-containing enzyme localized in peroxisomes and performs in various molecular and biochemical mechanisms. It is a key player in plant glycolate and glyoxylate accumulation pathways. The role of *GLO* in disease and stress resistance is well-documented in various plant species. Although studies have been conducted regarding the role of *GLO* genes from spinach on a microbial level, the direct response of *GLO* genes to various stresses in short-season and leafy plants like lettuce has not been published yet. The genome of *Lactuca sativa cultivar ‘Salinas’* (*v8*) was used to identify *GLO* gene members in lettuce by performing various computational analysis. Dual synteny, protein–protein interactions, and targeted miRNA analyses were conducted to understand the function of *GLO* genes. The identified *GLO* genes showed further clustering into two groups i.e., glycolate oxidase (GOX) and hydroxyacid oxidase (HAOX). Genes were observed to be distributed unevenly on three chromosomes, and syntenic analysis revealed that segmental duplication was prevalent. Thus, it might be the main reason for *GLO* gene diversity in lettuce. Almost all *LsGLO* genes showed syntenic blocks in respective plant genomes under study. Protein–protein interactions of *LsGLO* genes revealed various functional enrichments, mainly photorespiration, and lactate oxidation, and among biological processes oxidative photosynthetic carbon pathway was highly significant. Results of in-depth analyses disclosed the interaction of *GLO* genes with other members of the glycolate pathway and the activity of *GLO* genes in various organs and developmental stages in lettuce. The extensive genome evaluation of *GLO* gene family in garden lettuce is believed to be a reference for cloning and studying functional analyses of *GLO* genes and characterizing other members of glycolate/glyoxylate biosynthesis pathway in various plant species.

## Introduction

Lettuce (*Lactuca* *sativa L.*), is an annual, self-fertilizing, cool-season green leafy vegetable that belongs to the tribe Cicoreae and family Compositae, also called Asteraceae^1–4^. It requires an optimal temperature range of 13 °C to 18 °C and can be grown in regions with mild winter^5^. In genus *Lactuca,* cultivated lettuce (*Lactuca* *sativa L.*) is the only member grown commercial scale^6^. Horticulturally, lettuce cultivars with high consumer demand are classified based on the morphological differences. The cultivar under study, *L. sativa cv. salinas*, is marketed as Iceberg lettuce, also known as Crisphead lettuce, and has a large spherical head^7,8^. The genome sequence of *Lactuca sativa cultivar ‘Salinas’* has recently been released. Lettuce has a diploid genome with 2n = 2x = 18 chromosomes, and the genome size is estimated to be 2.5 Gb^9^. The hybrids of *L. sativa* are self-fertile^10^. Hydroponics is one of the most common production systems and addresses many issues regarding lettuce production^11–13^. Lettuce is nutrient-rich and contains potentially beneficial compounds such as vitamin A, antioxidants and minerals like calcium and iron. High levels of antioxidant compounds, namely polyphenols and vitamin C, as well as fiber determine the healing properties of lettuce^14^. It is demonstrated in a recent study that lettuce has beneficial effects on rats and humans for the prevention of cardiovascular diseases^15^. In an experiment, the internalization of human pathogens was demonstrated using lettuce as a model vegetable mainly because of its commercial importance^16^.

Glycolate oxidase (*GLO*) is important in plant photorespiration as the only enzyme that catalyzes glycolate oxidation into glyoxylate and hydrogen peroxide (H_2_O_2_) in peroxisomes^17,18^. Photorespiration is an essential pathway in plants for resistance against abiotic stresses, thus it supports plant growth in stress conditions, and it is caused by high light intensity, salinity and drought^19,20^. Hydroxy acid oxidase (*HAOX*) is quite similar to *GLO* and is mostly present in peroxisomes^21^ and because of the high similarity, this group is considered as a subgroup of *GLO* family^22^. *GLO* genes have been identified in many plant species such as *A. thaliana*, *N. benthamiana*, *S. oleracea*, and *O. sativa*. *GLO* family in *A. thaliana* and *S. oleracea* consists of five genes namely *GOX1*, *GOX2*, *GOX3*, *HAOX1* and *HAOX2*, whereas, rice genome contains only four functional *GLO* genes, Os*GLO1-4*, and *GLO* family of *N. benthamiana* consist of seventeen genes. Out of these seventeen genes, seven genes are identified as *NbGOXs* and ten as *NbHAOXs*, and it is the highest number of *GLO* genes ever reported in a plant^22–24^. The five genes in *A. thaliana GLO* family have high similarity among each other and have similar protein size that ranges from 364 to 374 amino acids. In rice, there is complexity in the interactions for *GLO* isozymes,which are controlled by *OsGLO1* and *OsGLO4*^24^.

A wide variety of physiological processes are regulated by *GLO* genes, such as the involvement of *GLO* genes in response against drought and salt stress^25,26^, and strongly regulate photosynthesis, possibly through Rubisco activase inhibition by feedback mechanism^27^. A *GLO* in rice has been shown to affect plant growth^28^, and the survival of maize in ambient air is dependent on the high activity of glycolate oxidase^29^. The reaction catalyzed by *GLO* produces glyoxylate and H_2_O_2_, which are known to be involved in plant disease resistance. Therefore, *GLO* supposedly may have crucial role in plant disease resistance^22,30^. Oxalate, biosynthesized by glyoxylate^31^, plays a positive role in the interaction between plants and pathogens such as *Sclerotinia sclerotiorum* and *Botrytis cinerea*^32–34^. Glyoxylate also has importance in biosynthesis of amino acids^35^. However, the pathway through which oxalate accumulates in plants and its regulation is independent of *GLO*^36^. H_2_O_2_, an important reactive oxygen species (ROS), plays a role as a signaling molecule in the interaction between plants and pathogens^37–40^. Nonetheless, there is direct evidence reported in *Arabidopsis*, *O. sativa*, and *N. benthamiana* that supports the role of *GLO* in plant disease resistance^22,23,41^.

Lettuce grown under low temperatures is associated with decreased fresh weight^42,43^. Quick changes in basic physiological processes can occur due to decreased ambient temperature^44^. Low temperature also results in reduced enzyme activity^45^. Freezing temperatures can cause blisters and peeling of leaves, leading to rot and decay, and can also act as sites of entrance for plant pathogens^5^. Drought and low temperature are factors that affect the cyclic electron flow (CEF) in plants^46^. Cyclic electron flow is pivotal for photorespiration, a mechanism initiated by glycolate oxidase, and helps the plant to resist abiotic stresses. A study revealed expression of *CBF/DREB1* genes in lettuce for resistance against low temperature (4 °C). However, it did not explain the action and function of glycolate oxidases under such a stressed environment.

Temperate leafy vegetables, when grown under hot climates, can develop a condition known as bolting. Bolting is a condition that occurs when a plant shifts from the vegetative to the reproductive stage in its lifecycle, but this doesn’t mean that bolting occurs in all plants. Iceberg lettuce is susceptible to bolting, whereas varieties such as summer crisp or French crisp, butter head and loose leaves are resistant to bolting^47^. High temperature promotes bolting, which results in rapid stem elongation, making lettuce bitter and unfit for marketing^48^. The activity of glycolate oxidases increased gradually during the vegetative stage, and it was maximum at 1st leaf stage of flowering in sunflower^49^. Research has been conducted to understand the mechanism and regulation of bolting in lettuce, but the activity of glycolate oxidases under its regulation is still unknown.

Heavy metal (Cd) soil contamination greatly affects lettuce growth and development^50^. Cadmium is easily absorbed by lettuce plants when grown in cadmium contaminated soils^1,51^. Cd stress reduces the contents of photosynthetic pigment in the leaf significantly and suppresses growth by decreasing the photosynthetic potential of the lettuce plant^52^. Light absorption is affected by leaf area; hence, leaf area is a critical factor in photosynthesis^53^. It has been seen that large and flat leaves show more photosynthetic activity than other leaf types^54^. The intensity of light is an important environmental factor that greatly affects the growth and development of a plant. Light acts as a source of energy and plant physiology adaptation is regulated by light^55^. Photosynthesis requiress photorespiration, and metabolic interaction exists between photosynthetic carbon fixation and photorespiration^56^. Studying the fluctuations in the expression of *GLO* genes under heavy metal (Cd) and light stress might provide a clear understanding of the role played by glycolate oxidases in improving lettuce tolerance to abiotic stresses.

Circular RNA (circRNA) is an RNA molecule through which transcriptional regulation of gene expression occurs in many species, but the functions of most circular RNAs are still unknown^57^. Targeting glycolate oxidase genes with light-responsive circRNAs might provide an understanding of light-regulated expression of *GLO* genes in lettuce.

*Bremia lactucae*, also known as the downy mildew of lettuce, is an oomycete known worldwide for causing devastating diseases in lettuce^58,59^. According to research, this pathogen caused disease in cultivated lettuce (*Lactuca sativa*), but wild lettuce (*Lactuca saligna*) resisted this pathogen^60^. The pathogen (*Bremia lactucae*) has been widely studied in *Lactuca serriola*, and it was found that disease severity and prevalence had close relationship with the type of habitat or size and density of populations (*L. serriola*)^58,61,62^. Since glycolate oxidase genes have been previously reported for strengthening plant resistance against various diseases, it would be interesting to show differential expression of glycolate oxidases in healthy and infected lettuce plants.

MicroRNAs, or simply miRNAs are important plant regulators that regulate various biological processes such as growth and development, defense against pathogens, and maintenance of proper internal conditions^63–66^. Among multiple species, miRNAs remain highly conserved, which means that the function of miRNA is very specific regardless of the species under observation. Targeted miRNA analysis can provide valuable insights into the expression and functions of glycolate oxidase genes in lettuce.

As mentioned earlier, glycolate oxidase enzyme is crucial for photorespiration, photorespiration goes simultaneously with photosynthesis, and photosynthetic inhibition occurs due to suppressing glycolate oxidase genes^67^. In C3 plants, glycolate oxidase is estimated to produce approximately 70% of the total respiratory hydrogen peroxide (H_2_O_2_)^68^, H_2_O_2_ acts as a key signaling molecule in stress-related signal transduction pathways^69^, and is widely known for its role in plant disease resistance^22,30^. Lettuce is considered one of the most important leafy vegetable crops in the world^2,70^, and like any other crop, profitable production depends on optimal photosynthesis and resistance to diseases and stresses. Lettuce is a suitable specimen for genetic studies due to its characteristic features, including a relatively short life cycle, high rate of natural self-pollination with full self-fertility, the possibility to carry out multiple crosses on one plant, and less space requirement for individual plant^4^. This research aimed to determine the role of glycolate oxidases in lettuce plant resistance to various biotic and abiotic stresses and to understand whether or not the results support the previously documented functions and significance of glycolate oxidases in various plant species.

## Results

### Identification of GLO gene family in *L. sativa*

To identify *GLO* genes in lettuce, the protein sequence of *SoGLO1*/Spo19861 (*S. oleracea*) retrieved from Spinachbase database was blast against the whole genome of lettuce (*Lactuca sativa v8/Lactuca sativa cv. salinas*). An initial analysis led to the identification of fourteen *GLO* proteins. Proteins encoded by the same gene isoforms and proteins containing truncated FMN-dependent dehydrogenase domain, were excluded from the analysis. Finally, five non-redundant *LsGLO* genes were identified and used for further analysis. The five proteins identified as glycolate oxidase in lettuce consisted of 1-3 *LsGOXs,* and 1-2 *LsHAOXs*.

### Identification of conserved domains in LsGLO genes

**Figure 1:**
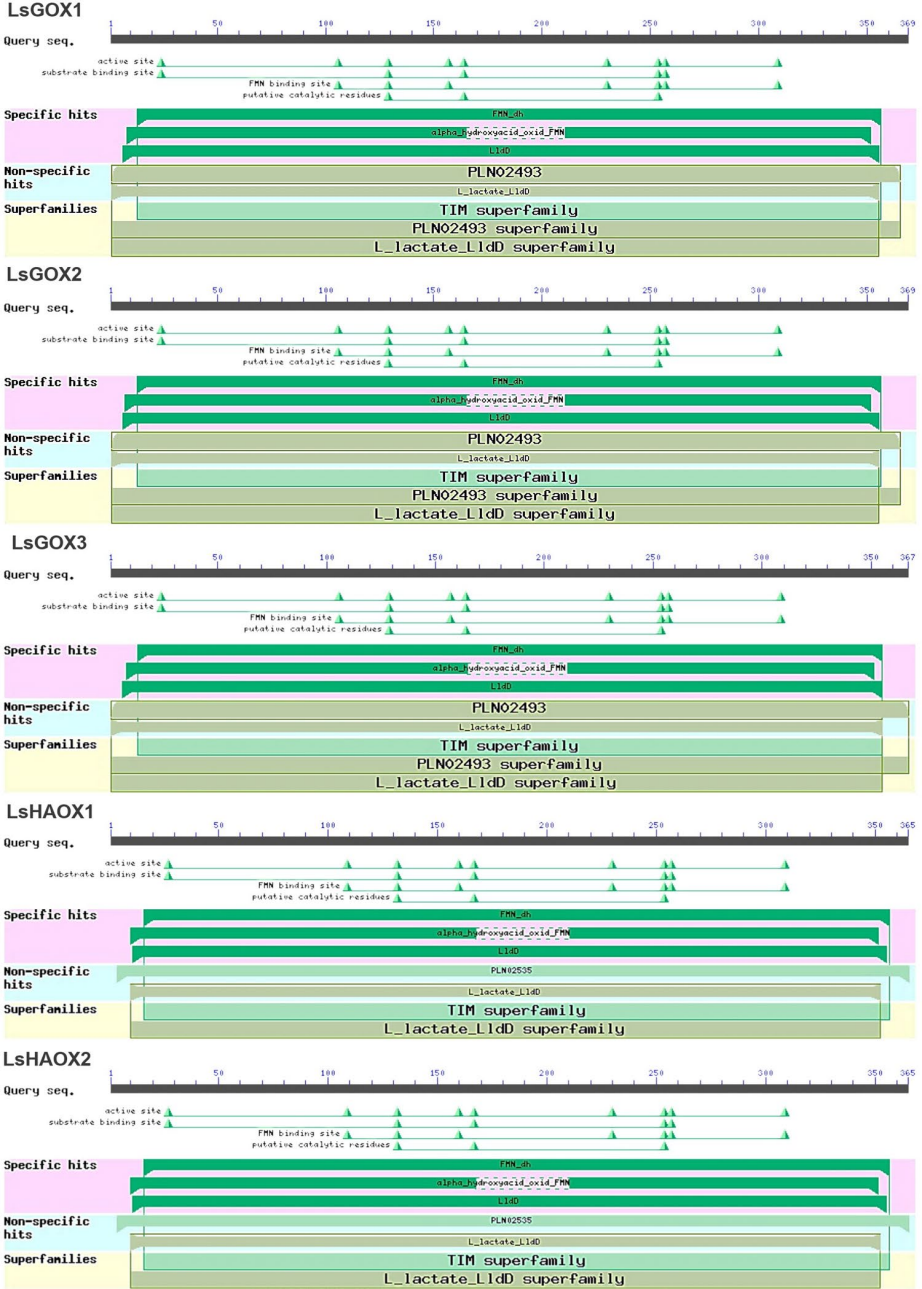
Identification of conserved domains in *LsGLOs* through NCBI CDD (https://www.ncbi.nlm.nih.gov/Structure/cdd/wrpsb.cgi) in comparison with *Arabidopsis GLO* genes.

The non-redundant *GLO* protein sequences from lettuce exhibit highly conserved domains, i.e., FMN-dependent dehydrogenase (FMN-dh), long chain alpha-hydroxy acid oxidase (alpha_hydroxyacid_oxid_FMN), and L-lactate dehydrogenase (LldD) were specific. Other non-specific domains were PLN02493, PLN02493 superfamily, PLN02535, L_lactate_LldD, and L_lactate_LldD superfamily, and these are typical features of glycolate oxidase proteins in general (Fig. 1, Supplementary material Table S1). Within the highly conserved sequence of alpha_hydroxyacid_oxid_FMN domain, there exists an amino acids gap from 165 to 210 in *LsGOXs*, and from 168 to 210 amino acids in *LsHAOXs*, and none of the protein had additional fragment in the middle of alpha_hydroxyacid_oxid_FMN domain sequence as reported in *N. benthamiana*. The same amino acids gap as *LsGOXs* was also observed in *AtGOXs*. A gap from 166 to 208 amino acids exists in *AtHAOXs* (Supplementary material Fig. S1). All 5 *LsGLO* proteins contain nine active sites, five substrate binding sites, seven FMN binding sites, and three putative catalytic residues. These sites were also reported in *A. thaliana* and *N. benthamiana* (Fig. 1).

### Physio-chemical properties of GLO genes and prediction of nuclear and sub-cellular localization signals

*LsGLO* genes encode proteins ranging from 365 to 369 amino acids with a molecular weight ranging from 40.2 to 40.6 kD. *LsHAOX1* and *LsHAOX2* are the smallest and *LsGOX1* and *LsGOX2* are the largest *GLO* proteins (Table 1). The isoelectric point (pI) of *LsGLO* proteins ranged from 8.7 to 9.1 (Table 1).

**Table 1:** Brief information about *LsGLO* genes.

GLO gene	Source Accession no	Chr no	Chr location (bp)	Direction	Size		pI	MW (kD)
					mRNA	Peptide (AA)		
LsGOX1	Lsat_1_v5_gn_9_103440	Lg9	165293651. 165299482	R	1110	369	8.8	40.6
LsGOX2	Lsat_1_v5_gn_5_120941	Lg5	244334988. 244338213	R	1110	369	9.0	40.5
LsGOX3	Lsat_1_v5_gn_4_29481	Lg4	42334516. 42337921	F	1110	367	9.1	40.2
LsHAOX1	Lsat_1_v5_gn_5_136840	Lg5	265492474. 265494942	R	1098	365	8.7	40.5
LsHAOX2	Lsat_1_v5_gn_5_136860	Lg5	265482601. 265487112	R	1098	365	8.7	40.4

Chr, chromosome; AA, amino acid sequence length; MW, molecular weight; pI, isoelectric point.

**Figure 2:**
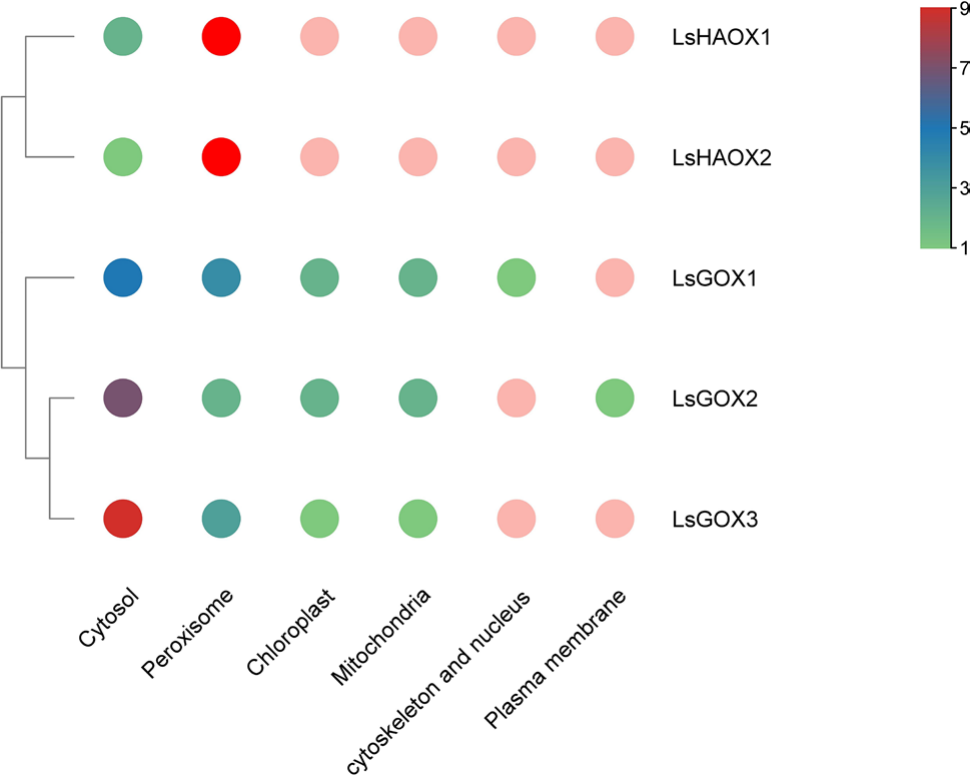
Heat map showing predicted subcellular localization of *LsGLO* proteins. Green indicates the lowest and red indicates the highest number of signals in *LsGLOs* displayed with hierarchy. ‘Cyto’, ‘Pero’, ‘Chlo’, ‘Mito’, ‘Plas’, and ‘Cysk_nucl’ represent cytosol, peroxisomes, chloroplast, mitochondria, plasma membrane, and cytoskeleton and nucleus.

No nuclear localization signals (NLS) were found in any of the lettuce *GLO* genes (Supplementary material Table S2). Sub-cellular location of lettuce *GLO* genes was predicted in cytoplasm, mitochondria, peroxisomes, and plastid etc., with *LsGOXs* being highly predicted in cytoplasm and *LsHAOXs* mainly in peroxisomes (Fig. 2, Supplementary material Table S2).

### Comparative phylogenetic analysis of GLO proteins

**Figure 3:**
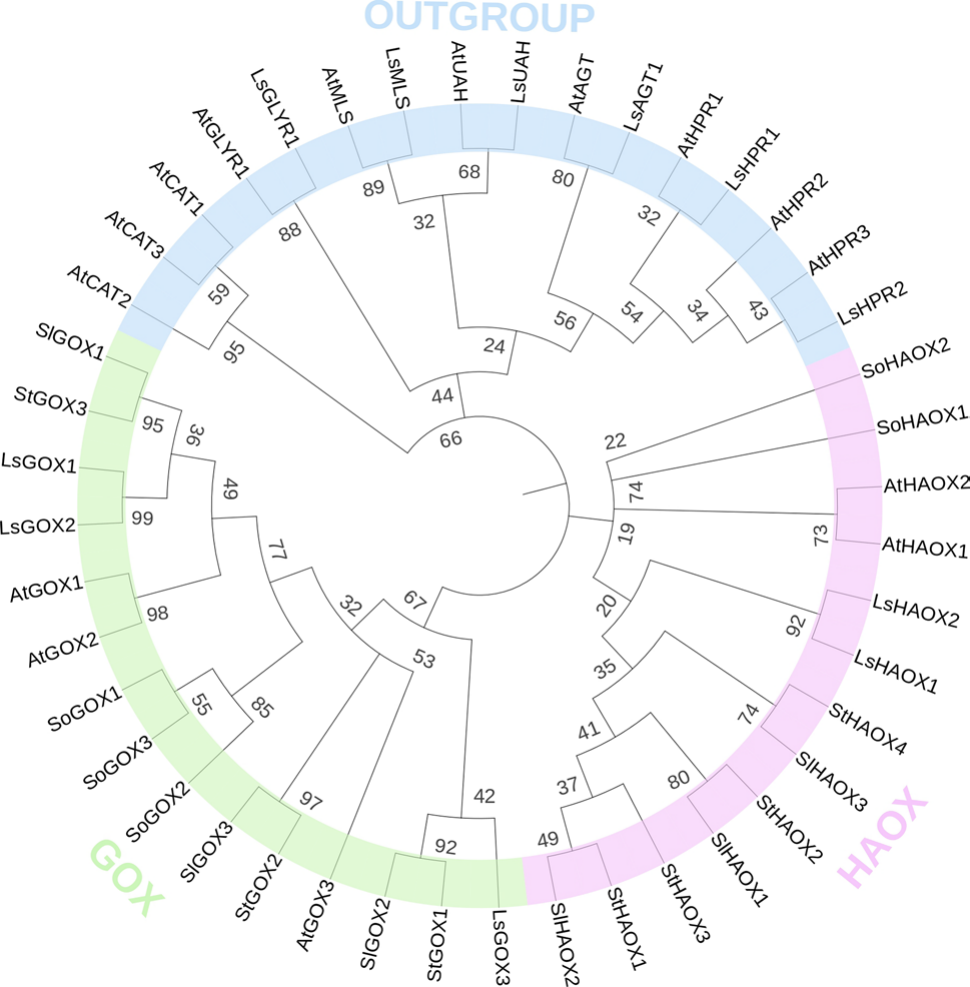
Phylogenetic relationships of *LsGLO* genes with other plant species( MEGA X v10.2.4). Using the UPGMA method to infer the evolutionary history with 1000 Bootstrap, and phylogenetic tree was constructed using an online tool, iTol v6 (Interactive Tree Of Life) (https://itol.embl.de). ‘At’, ‘So’, ‘Sl’, and ‘St’ represent *Arabidopsis*, *S. oleracea*, *S. lycopersicum*, and *S. tuberosum* genes, respectively. HPR, AGT, UAH, GLYR, MLS, and CAT were used as outgroups and represent hydroxypyruvate reductase, glyoxylate aminotransferase, ureidoglycolate amidohydrolase, glyoxylate reductase, malate synthase, and catalase, respectively. Different numbers indicate the bootstrap values on each node.

The phylogenetic analysis of *L. sativa GLO* genes was conducted compared with *S. oleracea*, *S*. *lycopersicum, S. tuberosum* and *Arabidopsis* GLO genes. An outgroup was added to create a more defined tree. To investigate the evolutionary relationships between *GLO* genes, Neighbor-Joining (NJ) phylogenetic tree was constructed through MEGA X v10.2.4 by aligning full-length protein sequences (Supplementary material Table S3). The result showed two groups that were named GOX and HAOX (Fig. 3). Group GOX consisted of total fifteen members in which three *GOX* proteins of *L. sativa*, *S. oleracea*, *S. lycopersicum*, *S. tuberosum,* and *Arabidopsis* were present. Group HAOX consisted of thirteen members in which two *HAOX* proteins of *L. sativa*, *S. oleracea,* and *Arabidopsis* were present along with three and four *HAOX* proteins of *S. lycopersicum* and *S. tuberosum*, respectively (Fig. 3). It was noticed that *LsGOX1* and *LsGOX2* genes and *LsHAOX1* and *LsHAOX2* genes were present in the same clade in their respective groups, however, *LsGOX3* was present in an entirely different clade in GOX group, and the same pattern was also observed in *Arabidopsis GLO* genes (Fig. 3). Also, *LsGOX1* and *LsGOX2* genes were present in the same clade with *AtGOX1* and *AtGOX2* in group GOX, whereas, *LsHAOX1* and *LsHAOX2* were present in a different clade from *AtHAOX1* and *AtHAOX2* in group HAOX (Fig. 3). All the *L. sativa GLO* genes showed close relationship with *S. lycopersicum* and *S. tuberosum GLO* genes (Fig. 3).

### Analysis of LsGLOs gene structure

**Figure 4:**
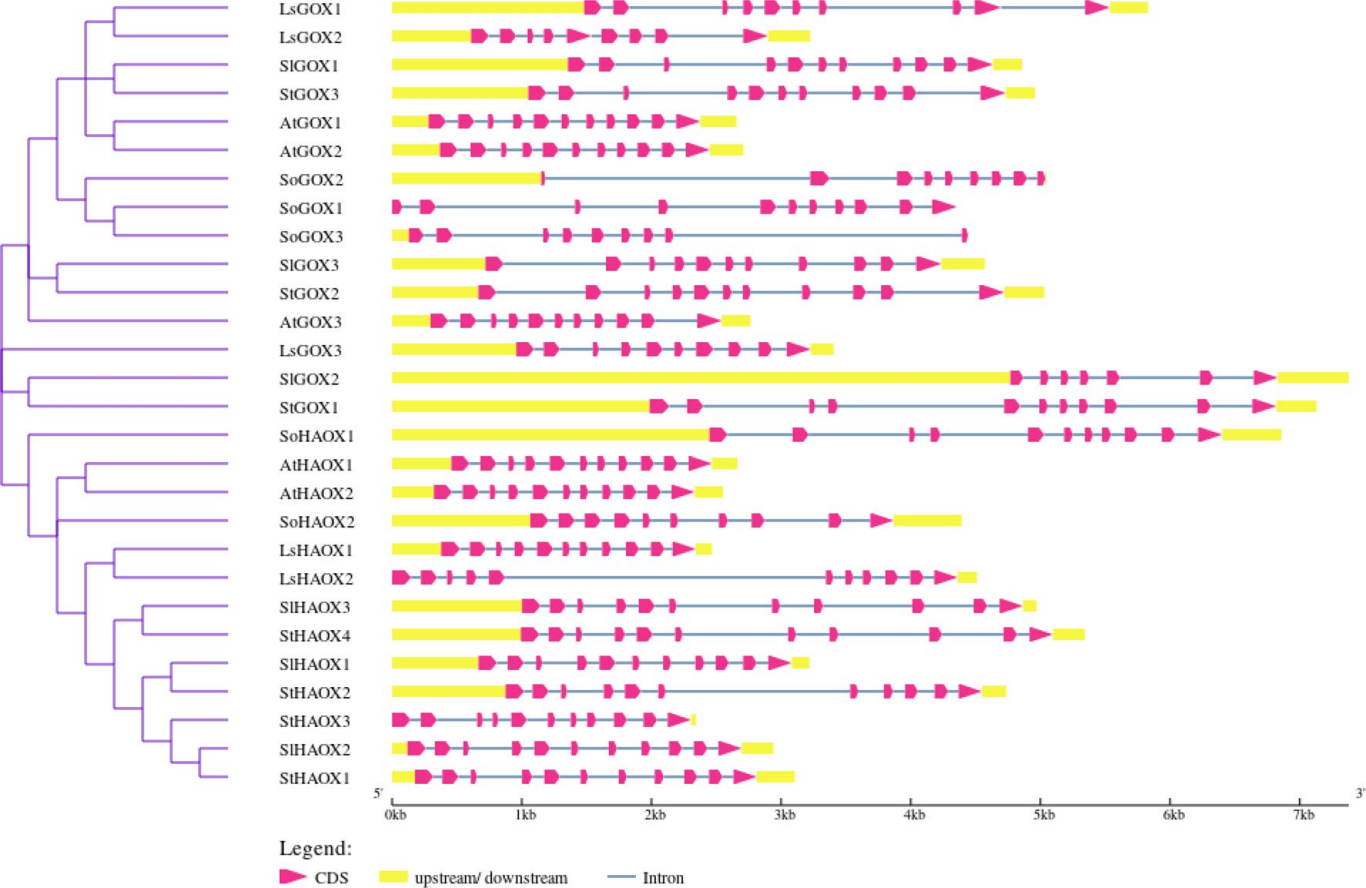
Gene structure of *L. sativa* (Ls) *GLO* genes compared to *S. oleracea* (So), *S. lycopersicum* (Sl), *S. tuberosum* (St), and *A. thaliana* (At) *GLO* genes. Pink arrows indicate exons, blue lines indicate introns, and purple lines represent phylogram (GSDS) at (http://gsds.cbi.pku.edu.cn/).

The organization of exon and intron provides the backbone of genes and can assist in studying evolutionary relationships between genes or organisms^71^. The number and distribution pattern of exon and intron are considered an evolutionary mark for a gene family. A comprehensive demonstration of the exon–intron structures of lettuce *GLO* genes and phylogeny analysis revealed that the gene structure pattern was consistent with the phylogenetic grouping (Fig. 4, Supplementary material Table S4). The *LsGOX* and *LsHAOX* genes showed similar gene structure patterns. An Almost similar number of introns and gene structure was displayed by Arabidopsis, spinach, tomato, and potato compared with lettuce (Fig. 4, Supplementary material Table S4). The number of introns varied from eight to ten in lettuce. *LsGOX1 and LsGOX3* genes possessed nine introns, *LsGOX2* possessed eight introns, and *LsHAOX1* and *LsHAOX2* included ten introns. All *GOX* genes contain eight to ten introns, except *SoGOX* genes, whereas all *HAOX* genes consist of ten introns, except *SoHAOX2* (Fig. 4).

### Identification of conserved motifs in *L. sativa* GLO genes

**Figure 5:**
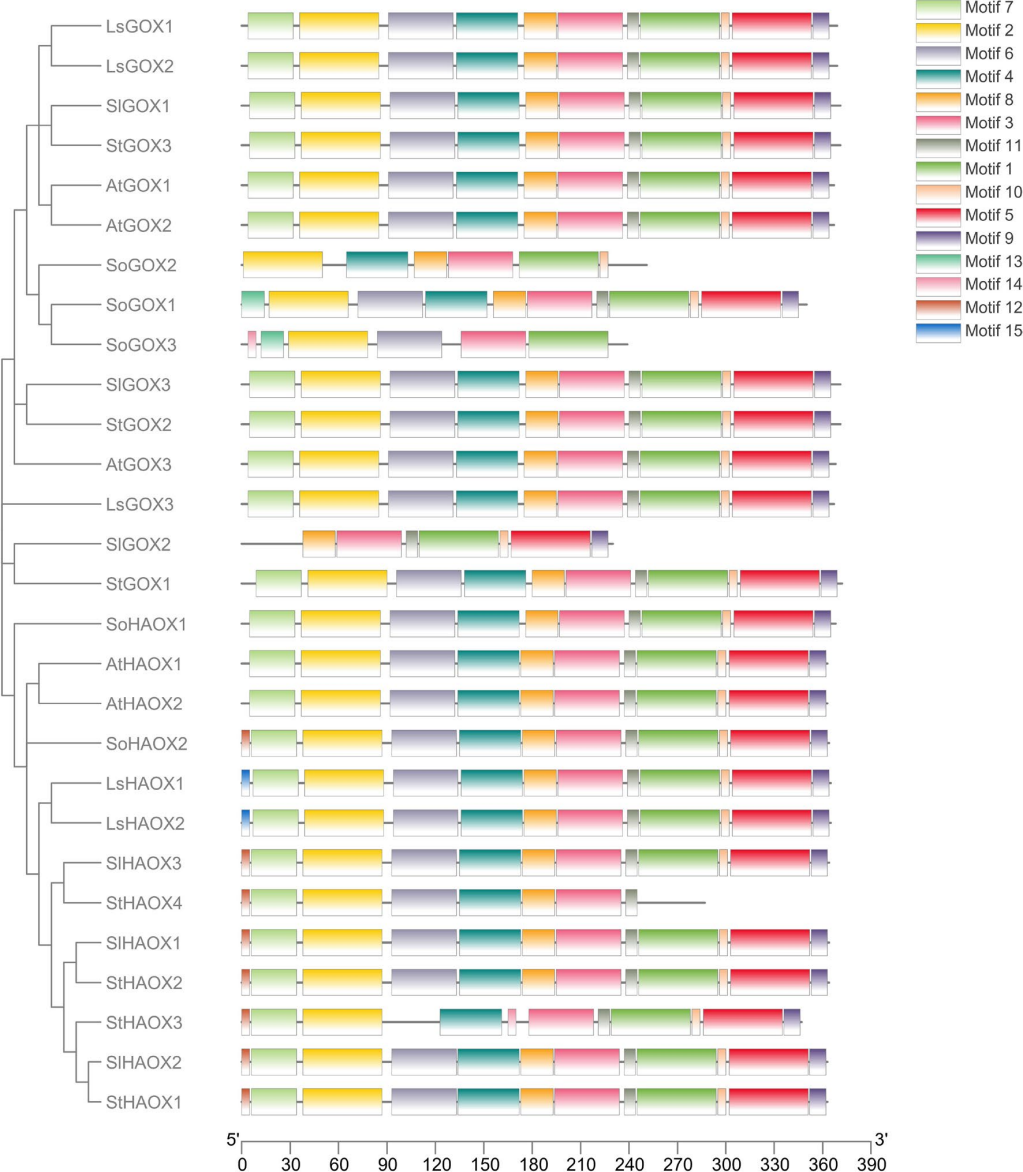
The distribution of 15 motifs in *GLO* proteins of various species. *GLOs* are displayed in hierarchy and ‘At’, ‘So’, ‘Sl’, and ‘St’ represent *Arabidopsis*, *S. oleracea*, *S. lycopersicum*, and *S. tuberosum GLO* genes, respectively. Bars with color gradient represent various motifs with a unique color code for each motif(TB tool v1.120).

The identification and distribution of fifteen motifs within all the *GLO* proteins were studied in *L. sativa*, *S. oleracea*, *S. lycopersicum*, *S. tuberosum,* and *Arabidopsis* using the MEME program (Fig. 5, Supplementary material Fig. S2, Supplementary material Table S5). The number and position of motifs were observed to be 100% conserved in all the *LsGLO* genes, except motif 15 which was present in only *LsHAOX* genes (Fig. 5). Similar to *LsHAOX* genes, motif 14 was present in the same position in most *S. lycopersicum*, *S. tuberosum* and *S. oleracea HAOX* genes. Glycolate oxidase genes in all species showed similar pattern except *S. oleracea* (Fig. 5).

### Identification of LsGLO orthologs in arabidopsis

Functional prediction of a gene through identifying characterized orthologs is extensively used as a powerful tool in computational biology^72,73^. Orthologous proteins in different species have been observed to show similar biological functions^73^. *LsGOX1* and *LsGOX2* showed maximum percentage homology with *AtGOX1*. *LsGOX1* showed 90.51, and *LsGOX2* showed 89.97 percentage homology with *AtGOX1*. *AtGOX1* was found to be expressed in cotyledon, fruit, leaf, guard cell, juvenile leaf during the seedling development stage, and non-host resistance, it regulates signal transduction pathway through reactive oxygen species (Table 2). *LsGOX3* expressed maximum percentage homology of 85.08 with *AtGOX3*. *AtGOX3* has previously shown expression in roots and mature to aging leaves during various developmental stages such as the flowering stage, differentiation, and expansion stage of the petal and embryo stage. *AtGOX3* metabolizes l-lactate to pyruvate when l-lactate is lower in concentration inside the cell (Table 2). *LsHAOX1* and *LsHAOX2* showed the highest homology of 75.55 and 74.01, respectively, with *AtHAOX1*. *AtHAOX1* was found to be expressed in seed and guard cells, and it encodes medium and long-chain hydroxy acid proteins as substrates (Table 2).

**Table 2:** Brief information about lettuce glycolate oxidase genes orthologs and its functions and expression in *Arabidopsis*.

*L. sativa* Gene ID	LsGOX1	LsGOX2	LsGOX3	LsHAOX1	LsHAOX2
*Arabidopsis* Ortholog Gene ID	AtGOX1, AtGLO1		AtGOX3, AtGLO5	AtHAOX1, AtGOX4, AtGLO4	
Accession no	AT3G14420		AT4G18360	AT3G14130	
% Homology	90.51	89.97	85.08	75.99	74.01
Functions	Regulating signal transduction pathway through ROS during nonhost resistance		Metabolize l-lactate to pyruvate at low intracellular concentrations	Encoding medium and long chain hydroxyl acid proteins as substrates	
Expression of Ortholog Gene					
Organs	Cotyledon, fruit, leaf, guard cell, juvenile leaf		Roots and mature to aging leaves	Seed and guard cells	
Stage	Seedling development stage		Flowering stage, petal differentiation and expansion stage and embryo stage		
Putative Functions of *Arabidopsis* Ortholog Gene	Photorespiration, defense response to bacterium, hydrogen peroxide biosynthetic process, oxidative photosynthetic carbon pathway		Photorespiration, innate immune response-activating signal transduction, oxidative photosynthetic carbon pathway	Photorespiration, defense response to bacterium, hydrogen peroxide biosynthetic process, oxidative photosynthetic carbon pathway	
References	23,105-107 (NCBI Ref Seq: NP_850585.2)		77,105,108,109	23,27,105,106,110,111	

### Chromosomal location and synteny analysis of lettuce GLO genes

**Figure 6:**
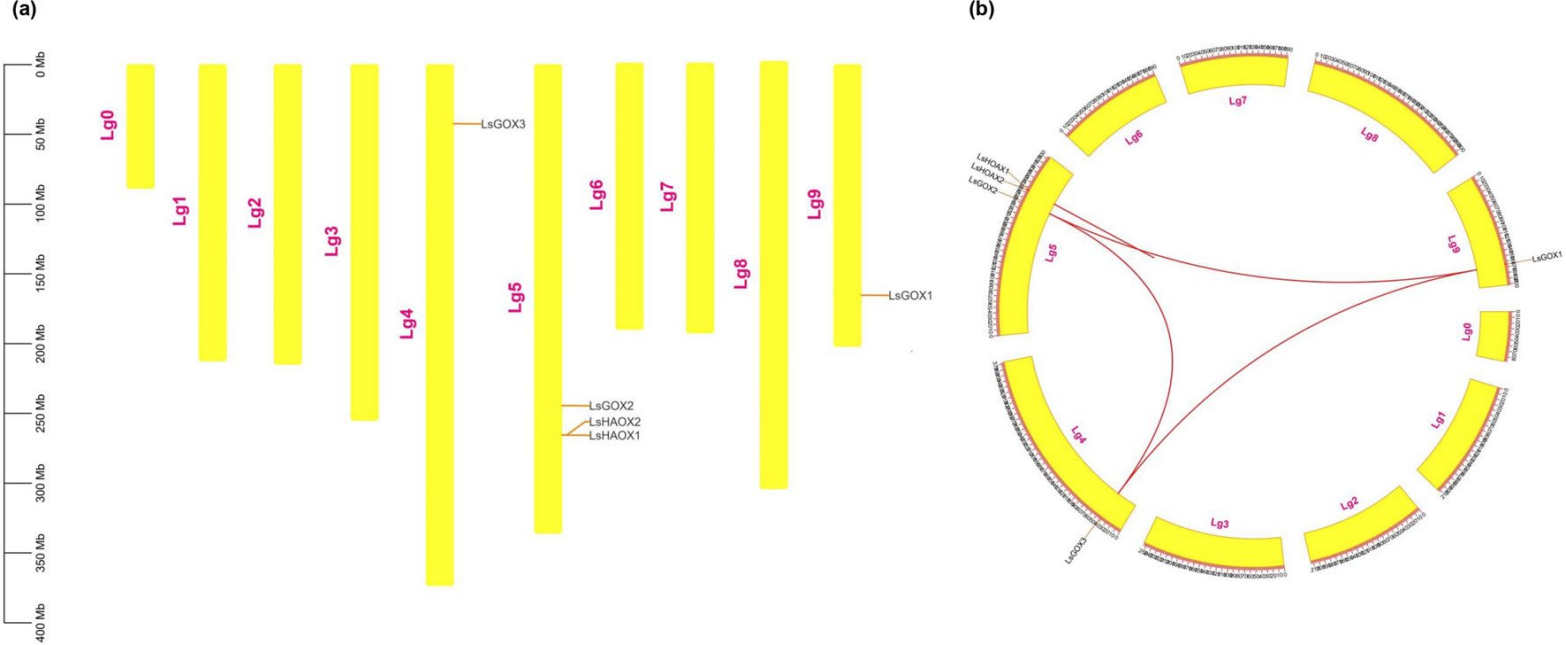
Distribution of *LsGLO* genes. (**a**) Position of *LsGLO* genes on chromosomes within the lettuce genome. (**b**) Syntenic relationship of *LsGLO* genes. Vertical (**a**) or curved (**b**) yellow blocks represent lettuce chromosomes, and labels on the right or top of each block represent the chromosome number. Rust colored lines represent the position of *LsGLO* genes on respective chromosomes, and red lines indicate segmental or tandem duplication of *LsGLO* genes(TB tool v1.120).

Chromosomal distribution analysis of *L. sativa GLO* genes demonstrated that out of ten lettuce chromosomes (Lg0-Lg9), *LsGLO* genes were present on only three chromosomes. The maximum of three *GLO* genes were located on Lg5, and these were *LsGOX2*, *LsHAOX1*, and *LsHAOX2*, whereas, *LsGOX1* and *LsGOX3* genes were found to be present on Lg9 and Lg4, respectively (Fig. 6). Furthermore, syntenic analysis was performed for *LsGLO* genes to gain insight about the probability of segmental or tandem duplication of *GLO* gene family in lettuce (Fig. 6). In *LsGLO* genes, three paralogous gene pairs were distributed non-uniformly in the lettuce genome, which suggested that they might be emerged from whole-genome duplication or segmental duplication, whereas, one paralogous gene pair located too close to each other on the same chromosome might have emerged as a result of tandem duplication. Synteny analysis revealed that *LsGOX1*, *LsGOX2*, and *LsGOX3* gene emerged as a result of the whole genome duplication (WGD) or segmental duplication, and *LsHAOX1*, and *LsHAOX2* genes emerged as a result of tandem duplication (Fig. 6).

### Dual synteny plot of lettuce GLO genes

**Figure 7:**
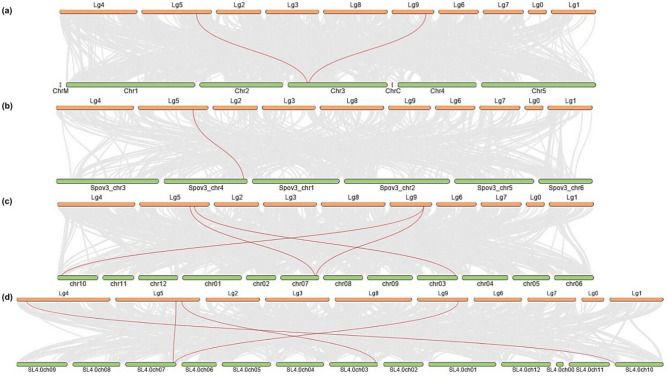
Dual synteny of *L. sativa GLO* genes with other plant species. Green bars represent *L. sativa* chromosomes, and orange bars represent chromosomes of (**a**) *Arabidopsis*, (**b**) *S. oleracea*, (**c**) *S. tuberosum*, and (d) *S. lycopersicum*. Labels on top or bottom of the bars represent chromosome number of the respective plant species. Syntenic *GLO* gene pairs are highlighted by red lines and gray lines in the background indicate other collinear blocks among respective genomes(TB tool v1.120).

To understand the evolution of *LsGLO* gene family, the syntenic relationship of *LsGLO* genes with *A. thaliana*, *S. oleraceae*, *S. lycopersicum,* and *S. tuberosum* was analyzed. Several *GLO* genes in *L. sativa* were revealed as orthologous genes in *A. thaliana*, *S. oleraceae*, *S. lycopersicum,* and *S. tuberosum* through collinearity analysis (Fig. 7, Table 3). Four *LsGLO* genes (80%) generally had syntenic relationships with GLO genes in other species. *LsGOX1* and *LsHAOX2* showed a syntenic relationship with *AtGOX2* and *AtHAOX1* on chromosome 3 (Chr3) in *Arabidopsis*. *LsHAOX2* showed synteny to Spov3_chr4.04560 on chromosome 4 (Spov3_chr4) in *S. oleraceae*. Syntenic relationship of *LsGOX1* and *LsGOX2* genes with *SlGOX1* on chromosome 7 (SL4.0ch07), *LsGOX3* with *SlGOX3* on chromosome 10 (SL4.0ch010), and *LsHAOX2* with *SlHAOX1* on chromosome 3 (SL4.0ch03) was observed in *S. lycopersicum*. Syntenic blocks were revealed in *LsGOX1* and *LsGOX2* with *StGOX3* on chromosome 7 (chr07), *LsGOX1* with *StGOX2* on chromosome 10 (chr10), and *LsHAOX2* with *StHAOX2* on chromosome 3 (chr03) in *S. tuberosum* (Fig. 7, Table 3). However, *LsHAOX1* did not show a syntenic relationship with any observed species.

**Table 3:** Collinear gene pairs of lettuce glycolate oxidase genes found in *S. oleracea*, *S. lycopersicum*, and *S. tuberosum* and *Arabidopsis* through dual synteny plot.

*L. sativa GLO* genes	*Arabidopsis*		*S. oleracea*		*S. lycopersicum*		*S. tuberosum*	
	Gene name	Chr no	Gene name	Chr no	Gene name	Chr no	Gene name	Chr no
*LsGOX1*	*AtGOX2*	Chr3	–	–	*SlGOX1*	SL4.0ch07	*StGOX3*	chr07
							*StGOX3*	
*LsGOX2*	–	–	–	–	*SlGOX1*		*StGOX2*	chr10
*LsGOX3*	–	–	–	–	*SlGOX2*	SL4.0ch10	–	–
*LsHAOX1*	–	–	–	–	*-*	–	–	–
*LsHAOX2*	*AtHAOX1*	Chr3	*Spov3_chr4.04560*	Spov3_chr4	*SlGOX3*	SL4.0ch03	*StHAOX2*	chr03

### Evaluation of duplication event of lettuce GLO genes

**Figure 8:**
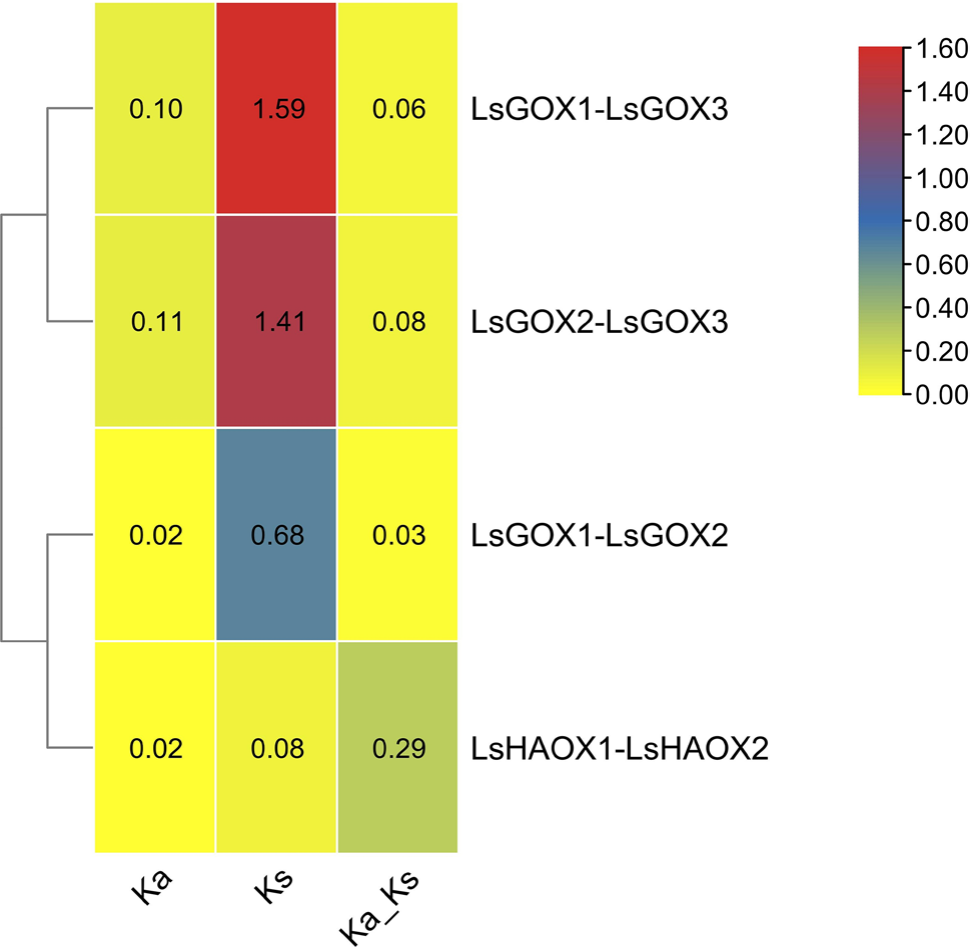
Ks and Ka values of *L. sativa GLO* genes. Color gradient represents values, yellow represents the lowest and red represents the highest value, displayed in hierarchy.

The values of Ks, Ka, and Ka/Ks ratio of *LsGLO* genes were estimated through TBtools using simple Ka/Ks calculator (Fig. 8). Ks depicts the number of synonymous substitutions per synonymous site, whereas Ka shows the number of nonsynonymous substitutions per nonsynonymous site and the ratio of nonsynonymous (Ka) versus synonymous (Ks) mutation was represented by Ka/Ks. This ratio ranged from 0.03 in *LsGOX1*/*LsGOX2* pair to 0.29 in *LsHAOX1*/*LsHAOX2* pair (Fig. 8). All four paralogous pairs in lettuce had Ka/Ks ratio less than 0.3 which suggested the probability of limited functional divergence in the duplication process due to purifying selection.

### Analysis of LsGLOs cis-regulatory elements

**Figure 9:**
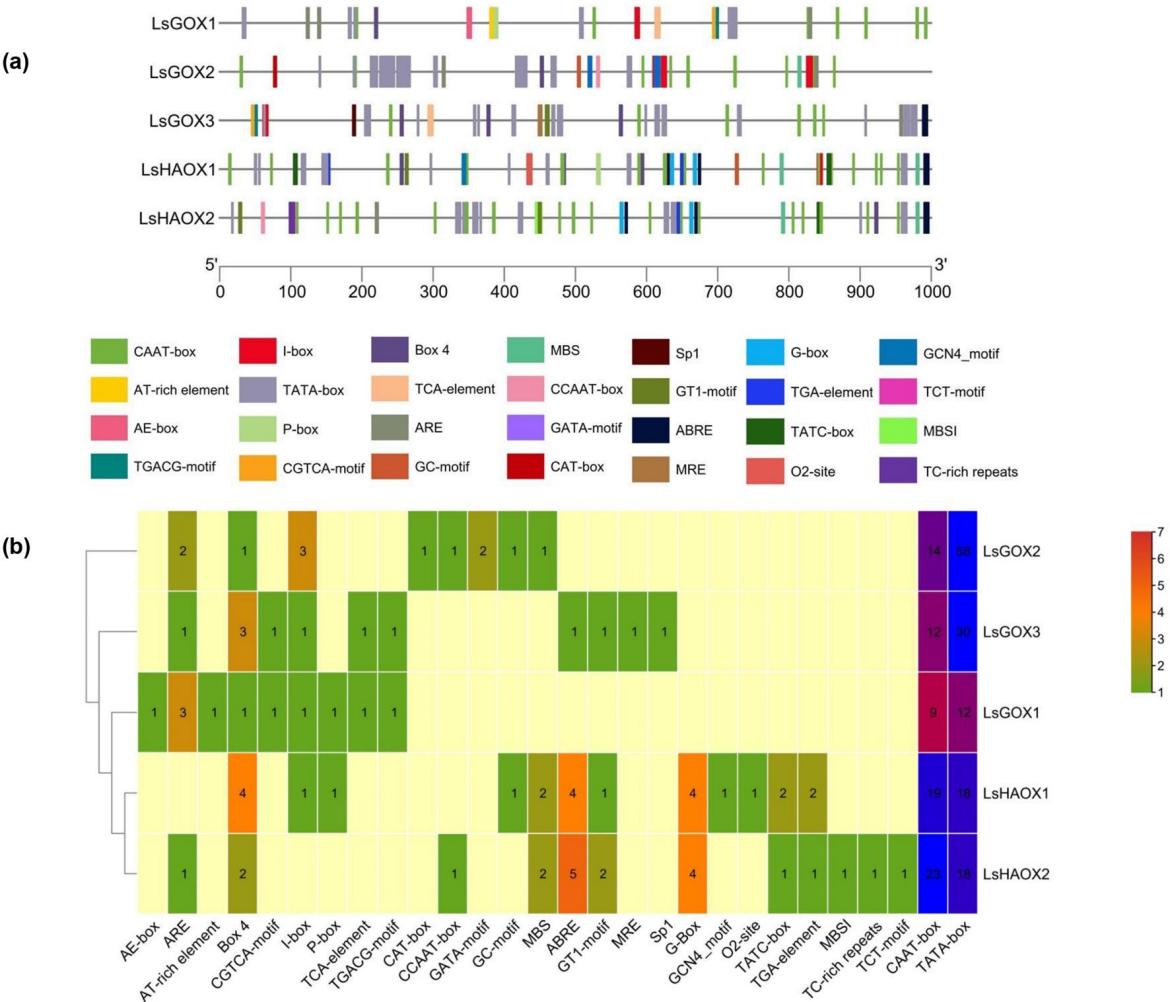
Identification of cis-acting elements in *LsGLO* genes. (**a**) Position of various cis-acting elements on the promoter region of *LsGLO* genes is represented on a scale of 1000 bps. Colored legends at the bottom represent various cis-elements found in each *LsGLO* gene, and each element is represented by a specific color. (**b**) Heat map representing the number of each cis- element found in *LsGLO* genes via a color gradient from green, representing the lowest, to red, representing the highest number of occurrences, and beige represents the absence of these elements.

The presence and organization of various *cis*-regulatory elements present on the promoter region at the binding site of transcription factors affect the spacio-temporal transcriptomic expression of the genes. Therefore, to evaluate the putative functions of *LsGLO* genes, an *in-silico* analysis was conducted through the PlantCare database. Lettuce *GLO* genes consisted of light responsive, endosperm specific, hormone specific, meristem specific, metabolism related, and stress and defense related cis-regulatory elements (Fig. 9, Supplementary material Table S6). *GLO* family in lettuce contained nine cis-regulatory elements that are responsive to light, these were AE-box, Box 4, GATA-motif, I-box, GT1-motif, MRE, Sp1, G-box, and TCT-motif. Box 4 was present in all *LsGLOs*, and I-box was in all *LsGLOs*, except, *LsHAOX2*. GT1-motif was present in *LsGOX3*, *LsHAOX1* and *LsHAOX2*. G-box was present in *LsHAOX1* and *LsHAOX2* only. AE-box was only present in *LsGOX1*, GATA-motif was present only in *LsGOX2*, MRE and Sp1 were present in *LsGOX3* only, and TCT-motif was solely present in *LsHAOX2* (Fig. 9, Supplementary material Table S6).

Three defense related cis-regulatory elements in *LsGLOs* were CGTCA-motif, TGACG-motif, and TCA-element. CGTCA-motif and TGACG-motif are involved in response to methyl jasmonate and TCA-element is involved in response towards salicylic acid as a result of biotic stress, and these elements were limited to *LsGOX1* and *LsGOX2*. Abiotic stress-related four cis-regulatory elements, ARE, GC-motif, MBS, and TC-rich repeats, were also present in *LsGLOs*. ARE, which is essential for anaerobic induction, was absent only in *LsHAOX1*, GC-motif, which is involved in anoxic specific inducibility, was present only in *LsGOX2* and *LsHAOX1*, MBS, which is involved in the induction of drought, was present in *LsGOX2*, *LsHAOX1,* and *LsHAOX2*. TC-rich repeats involved in response to stress and defense were limited to *LsHAOX2* (Fig. 9, Supplementary material Table S6).

GCN4_motif, which is involved in endosperm expression was present only in *LsHAOX1*, and CAT-box, which is related to meristem expression was present only in *LsGOX2*. O2-site involved in regulation of zein metabolism and MBSI involved in the regulation of flavonoid biosynthetic genes, was present only in *LsHAOX1* and *LsHAOX2*, respectively. AT-rich element, which acts as the binding site of AT-rich DNA binding protein (ATBP-1), was present only in *LsGOX1*, and CCAAT-box, which acts as MYBHv1 binding site, was present in *LsGOX2* and *LsHAOX2*. TATA-box and CAAT-box which are highly common cis-regulatory elements, were present in all lettuce *GLO* genes (Fig. 9, Supplementary material Table S6). The cis-regulatory elements identified among five *LsGLOs*, and their functional annotation, are shown in Supplementary material Table S6.

### Analysis of protein–protein interaction network

**Figure 10:**
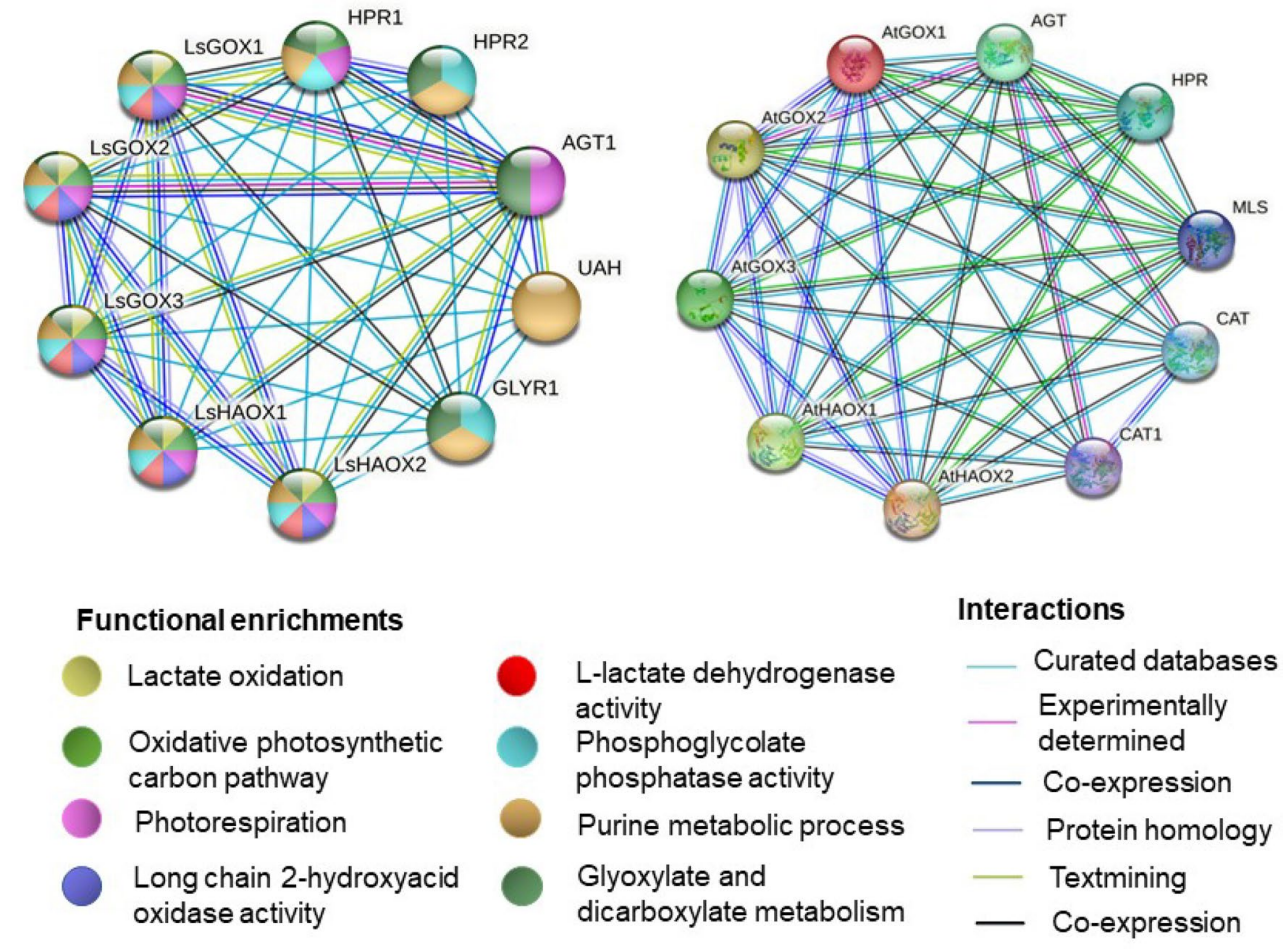
Protein–protein interactions of *LsGLO* proteins predicted through STRING database(https://string-db.org). (**a**) Each colour represents a highly significant GO enrichment and type of interaction identified within the network. (**b**) Comparison of protein–protein interacting structure in *L. sativa* (Ls) and *A. thaliana* (At). HPR, AGT, UAH, GLYR, MLS, and CAT represents hydroxypyruvate reductase, glyoxylate aminotransferase, ureidoglycolate amidohydrolase, glyoxylate reductase, malate synthase, and catalase, respectively.

Interaction among proteins reflects various important plant functions and processes such as signal transduction pathway, plant developmental, physiological, and pathological processes^74^. In lettuce *GLOs*, protein–protein interaction was studied through STRING database. Glycolate oxidase proteins showed interaction among various *GLO* proteins and with various other proteins in *Lactuca sativa* genome (Fig. 10).

The STRING database provided various functional enrichments such as molecular functions, biological processes, and KEGG pathway etc. present in LsGLOs proteins (Table 4). Biological processes related to lactate oxidation, photorespiration, and oxidative photosynthetic carbon pathway showed the highest significance at *p*-value of 3.48 × 10^−13^, 2.08 × 10^−14^, and 2.39 × 10^−15^, respectively. Molecular functions that showed high enrichment are L-lactate dehydrogenase activity and long-chain 2-hydroxy acid oxidase activity at *p*-value of 4.15 × 10^−13^. Phosphoglycolate phosphatase activity and purine metabolism were identified as significantly enriched GO terms in local network cluster (STRING) at *p*-value of 1.82 × 10^−20^ and 5.16 × 10^−21^, respectively. KEGG signaling pathway revealed glyoxylate and dicarboxylate metabolism as most enriched functional category at *p*-value of 4.82 × 10^−18^. These significantly enriched GO terms showed presence in all *LsGLO* protein–protein interactions (Fig. 10, Table 4). Hydrogen peroxide biosynthetic process (biological process) and glycolate oxidase activity (molecular function) were specific to only *LsHAOXs* and *LsGOXs*, respectively (Table 4). Interestingly within the local string network cluster, butanoate metabolism showed presence only in *LsGOX1* and *LsGOX3* (Table 4).

**Table 4:** List of functional associations of lettuce GLO proteins predicted through protein–protein interactions.

Gene ID	LsGOX1	LsGOX2	LsGOX3	LsHAOX1	LsHAOX2
Molecular Functions	2-hydroxy acid oxidase activity, oxidoreductase activity, nucleotide binding, *glycolate oxidase activity	2-hydroxy acid oxidase activity, oxidoreductase activity, nucleotide binding, *glycolate oxidase activity	2-hydroxy acid oxidase activity, oxidoreductase activity, nucleotide binding, *glycolate oxidase activity	2-hydroxy acid oxidase activity, oxidoreductase activity, nucleotide binding	2-hydroxy acid oxidase activity, oxidoreductase activity, nucleotide binding
Biological Processes	Photorespiration, lactate oxidation	Photorespiration, lactate oxidation	Photorespiration, lactate oxidation	Photorespiration, lactate oxidation, *hydrogen peroxide biosynthetic process	Photorespiration, lactate oxidation, *hydrogen peroxide biosynthetic process
KEGG Pathway	Glyoxylate and dicarboxylate metabolism, carbon metabolism, biosynthesis of secondary metabolites	Glyoxylate and dicarboxylate metabolism, carbon metabolism, biosynthesis of secondary metabolites	Glyoxylate and dicarboxylate metabolism, carbon metabolism, biosynthesis of secondary metabolites	Glyoxylate and dicarboxylate metabolism, carbon metabolism, biosynthesis of secondary metabolites	Glyoxylate and dicarboxylate metabolism, carbon metabolism, biosynthesis of secondary metabolites
Local String Network Cluster	Purine nucleobase metabolic process, phosphoglycolate phosphatase activity, *glyoxylate bypass, *butanoate metabolism	Purine nucleobase metabolic process, phosphoglycolate phosphatase activity, *glyoxylate bypass	Purine nucleobase metabolic process, phosphoglycolate phosphatase activity, *glyoxylate bypass, *butanoate metabolism	Purine nucleobase metabolic process, phosphoglycolate phosphatase activity	Purine nucleobase metabolic process, phosphoglycolate phosphatase activity

Asterisk (*) represent group specific activities.

Partner proteins, namely hydroxy pyruvate reductase (HPR), alanine glyoxylate aminotransferase (AGT), ureidoglycolate amidohydrolase (UAH), and glycolate reductase (GLYR1) showed presence in the protein–protein interaction network other than lettuce *GLO* proteins (Fig. 10, Table 4). HPR1 revealed interactions such as co-expression with *LsGOX1* and *LsGOX2* (Fig. 10). HPR2 and UAH showed only known interactions from curated databases with all *LsGLO* genes. AGT1 showed co-expression with all *LsGLO* genes and gene co-occurrence with *LsGOX1* and *LsGOX2*. GLYR1 represented co-expression with *LsGOX1* and *LsGOX2* (Fig. 10). The genes representing these proteins in *L. sativa*, its ortholog, and the functions of these orthologs in *Arabidopsis thaliana* were provided in Table 5.

**Table 5:** List of functional proteins predicted as *LsGLO* protein partners through STRING database.

Partner proteins in *L. sativa*		Functions in *Arabidopsis thaliana*	
Protein name	Gene ID	Gene ID	Functions
HPR1	Lsat_1_v5_gn_3_46860	AT1G68010	During photorespiration it catalyzes the conversion of hydroxypyruvate (HP) into glycerate, a NADPH-dependent reaction. Helps in seed germination by facilitating the oxidation of fatty acids in the absence of malate dehydrogenase
HPR2	Lsat_1_v5_gn_4_102800	AT1G79870	Provides cytosolic bypass during photorespiration by catalyzing NADPH-dependent reduction of glyoxylate and hydroxypyruvate (HP) into glycolate and glycerate in the cytoplasm
AGT1	Lsat_1_v5_gn_5_64980	AT2G13360	It catalyzes transamination reactions as a photorespiratory enzyme by utilizing various substrates such as asparagine. It is a catabolic enzyme exclusively in asparagine synthetase (ASN) metabolism. During seedling germination, it is involved in the development of roots through regulation of serine homeostasis and conversion of acetate after seed germination
AAH-2	Lsat_1_v5_gn_5_72961	AT5G43600	Facilitates the complete degradation of purine nucleotides by working in a sequence with UGLYAH and UAH without producing urea as an intermediate product
GLYR1	Lsat_1_v5_gn_5_176021	AT3G25530	Catalyzes NADPH-dependent reduction reaction involves converting glyoxylate into glycolate and succinic semialdehyde (SSA) to gamma-hydroxybutyrate in vitro. Inducing tolerance in plant against oxidative stress through conversion of glyoxylate in glycolate metabolism and catalysis of succinic semialdehyde in gamma-aminobutyric acid (GABA) metabolism. The non-protein amino acid (GABA) accumulates in plant tissues as a result of either abiotic or biotic stress and regulates plant growth

Comparing the protein–protein interaction of *A. thaliana* and *L. sativa* glycolate oxidase proteins revealed interesting results (Fig. 10). *A. thaliana GLO* genes showed complex interaction with other proteins compared to *L. sativa*. Contrary to *L. sativa GLO* genes mainly showing co-occurrence and gene co-expression with AGT1 and GLYR1, *A. thaliana GLO* genes showed co-expression with HPR (AT1G68010.2), AGT (AT2G13360.1), CAT (catalase) (CAT1/AT1G20630.1, CAT2/AT4G35090.1), and MLS (malate synthase/AT5G03860.1) (Fig. 10). Catalase occurs mainly in all aerobically respiring organisms and plays a role in protecting cells from the toxic effects of hydrogen peroxide by reducing hydrogen peroxide into water and oxygen using heme group as cofactor (TAIR).

### Expression analysis of LsGLO genes in various organs under stress environment

Differential expression patterns of all *LsGLOs*, when exposed to various biotic, abiotic and developmental stresses, were analyzed using the available lettuce expression data on NCBI GEO. These expression data include the following stresses; low temperature, heavy metal (Cd), light, bolting, over-expression of LsAP2, and *Bremia lactucae* infection.

### Low temperature (4 °C) stress affecting LsGLO genes

**Figure 11:**
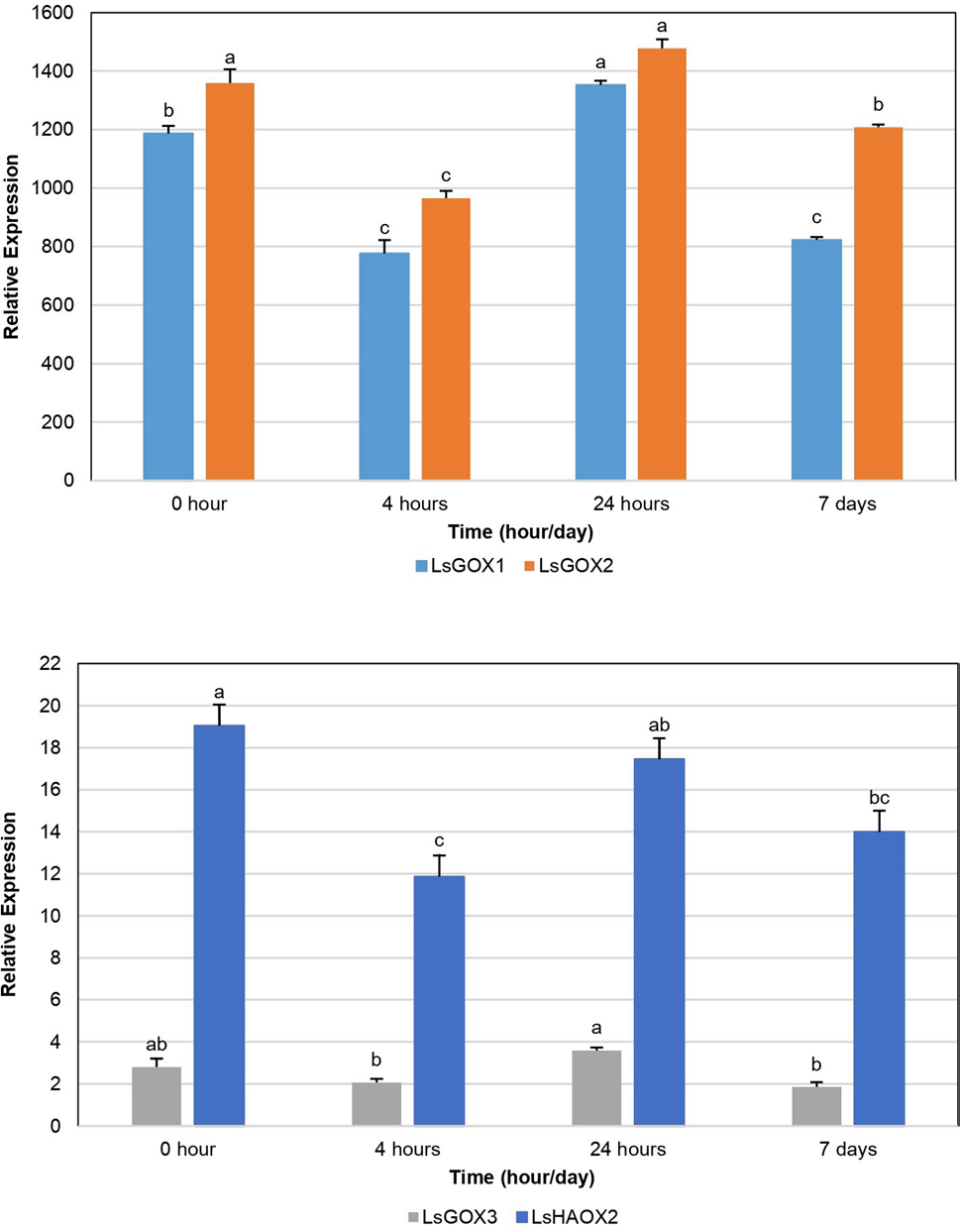
Expression of lettuce *GLO* genes under low temperature (4 °C) stress. The expression in control group (20 °C/0 h) and experimental group (exposure to 4 °C for 4 h, 24 h, and 7 days) lettuce plants was analyzed through multiple comparisons of means using Tukey HSD test at a significance level of 0.05. Different letters indicate significant differences and error bars indicate standard error of 3 replicates. *LsGLO* genes are represented by different colours as shown in legends.

When the lettuce plant was exposed to low temperature (4 °C) for 0 h, 4 h, 24 h and 7 days, all *LsGLO* genes showed statistically significant (*p* < 0.05) results in above-ground part of lettuce, except *LsHAOX1*. The expression level was quite high for *LsGOX1* and *LsGOX2* and very low, almost negligible, for *LsGOX3* and *LsHAOX2* (Fig. 11). The graph showed that initially, the expression of *LsGOX1* and *LsGOX2* gene decreased rapidly when lettuce plants were exposed to 4 °C temperature for 4 h as compared to the control group (0 h) (Fig. 11). The expression of these genes then started to increase and reached its peak after 24 h. At this point, the expression level of *LsGOX1* and *LsGOX2* exceeded the expression in the control group (Fig. 11). The expression, then, started to decrease, and after 7 days it was lower than the control group but still higher than expression of *LsGLOs* in 4 h group (Fig. 11).

### Gene expression during various developmental stages in bolting-sensitive and bolting-resistant lines of *L. sativa*

**Figure 12:**
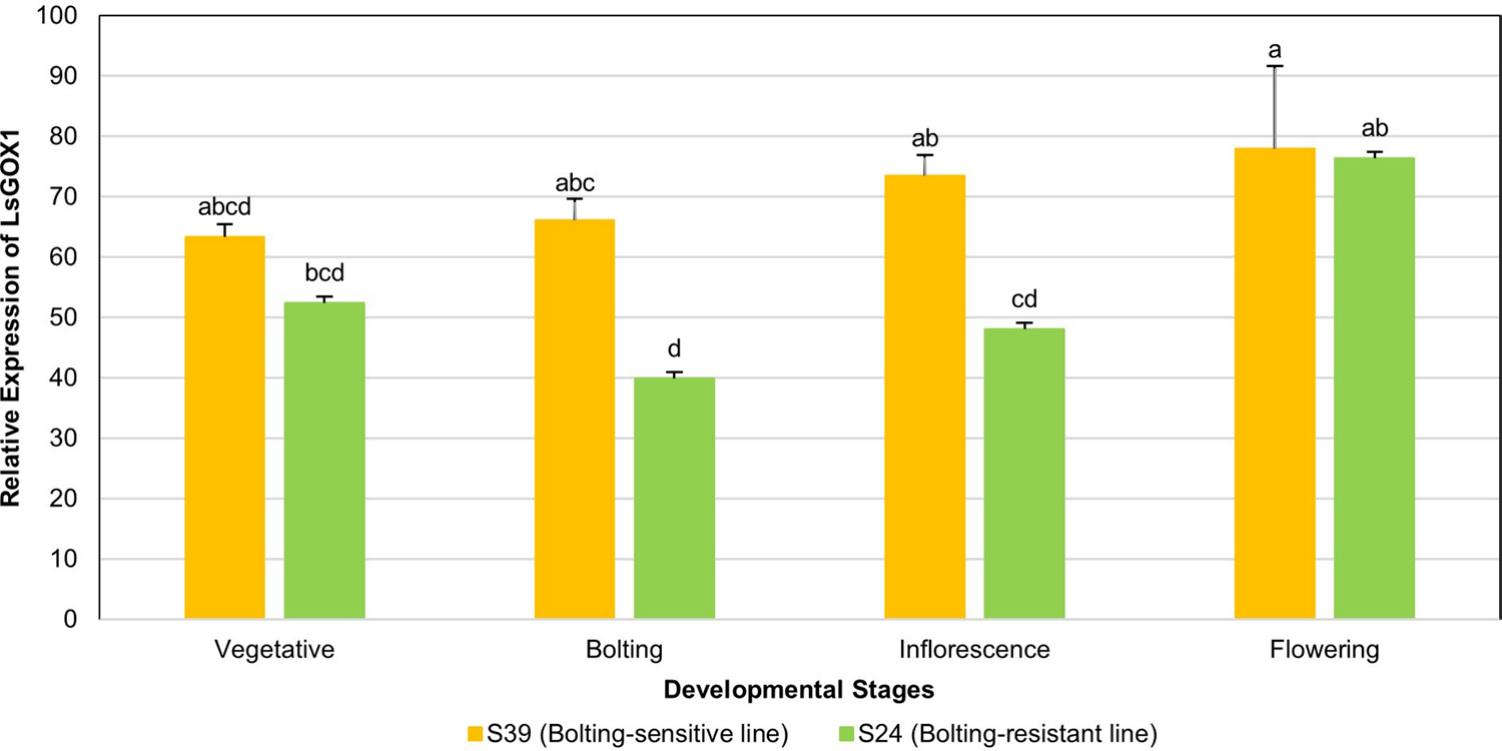
Expression of *LsGOX1* under bolting stress in in bolting-sensitive S39 and bolting resistant S24 lettuce lines. Different letters indicate significant differences and these differences were determined by Tukey HSD test at a significance level of 0.05 and error bars indicate standard error in biological replicates.

The expression data generated to study the regulation of bolting in lettuce showed the expression of all *LsGLO* genes except *LsHAOX2*, however, only the expression showed by *LsGOX1* was statistically significant (*p* < 0.05). It was observed that high expression was given by *LsGOX1* in SAM cells of bolting-sensitive line S39 as compared to bolting-resistant line S24 of lettuce (Fig. 12). In bolting-sensitive line S39, the expression of *LsGOX1* increased gradually in all four stages and reached a maximum level of expression at flowering stage (Fig. 12). However, in bolting-resistant line S24 of lettuce, the expression of *LsGOX1* showed sudden decrease initially in bolting stage from the vegetative stage, but then gradually increased from bolting stage and reached its peak at flowering stage (Fig. 12). Collectively, the flowering stage showed the highest expression of *LsGOX1*, and difference in expression of *LsGOX1* was highest at bolting and inflorescence stage in both bolting-sensitive line S39 and bolting-resistant line S24 of lettuce (Fig. 12).

### Differential expression of LsGLO genes in leaves and roots of *L. sativa* under cadmium (Cd) stress

**Figure 13:**
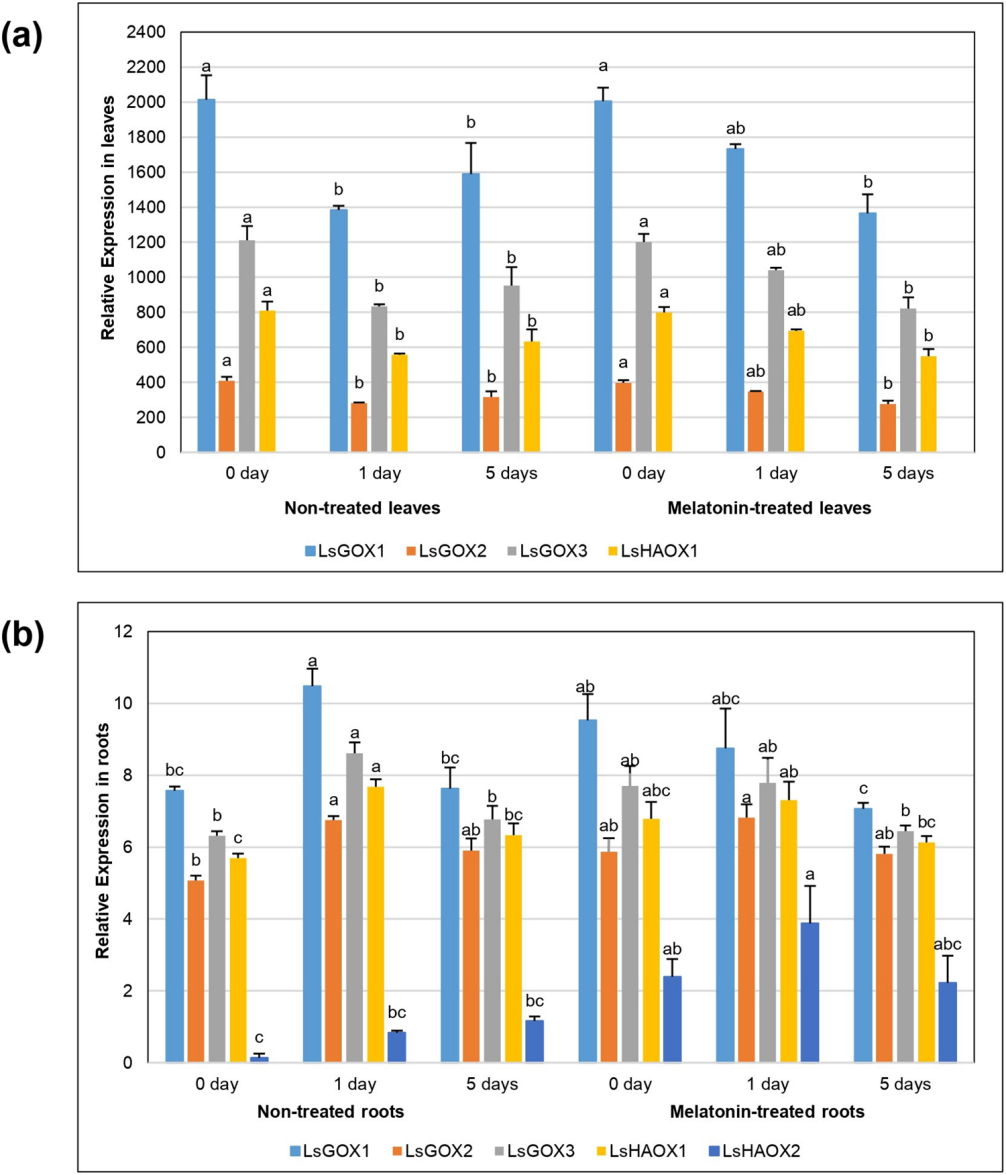
Expression pattern of *LsGLO* genes under cadmium (Cd) stress. (**a**) Expression in treated and non-treated lettuce leaves. (**b**) Expression in treated and non-treated lettuce roots. 0, 1, and 5 represent duration of cadmium stress in days. Error bars indicate standard error in biological replicates. Significant differences in expression of *LsGLO* genes were determined by Tukey HSD test (*p* < 0.05), and the differences are indicated by letters a, b, and c. *LsGLO* genes are represented by different colours as shown in legends.

In expression profiling of Italian lettuce (*Lactuca sativa L. var. ramose Hort*.) where melatonin treated and non-treated groups were grown under cadmium (Cd) stress for 0 day (control group), 1 day, and 5 days, all *LsGLO* genes showed statistically significant expression (*p* < 0.05) in roots and leaves, except, *LsHAOX2* in leaves. The expression of *LsGLO* genes in leaves was significant than the expression in roots (Fig. 13).

In leaves, melatonin-treated, and non-treated group showed the same level of expression in the control group (0 day), then at day 1 the expression of *LsGLO* genes was significantly reduced in both groups, but melatonin treated group showed higher expression than non-treated group, however at day 5, the expression of all *LsGLO* genes was lower in melatonin treated group than non-treated group. At day 5, expression in both groups was relatively higher than in day 1 (Fig. 13).

In roots, the control group (day 0) showed higher expression of GLO genes in the melatonin-treated group than the non-treated group. At day 1, the expression level of *LsGLO* genes in non-treated roots was comparatively high than control group, however, melatonin-treated roots showed decrease in expression level from control (day 0) (Fig. 13). At day 5, non-treated group showed same level of expression as a control group; however, expression of melatonin-treated group was lower than the control group (0 day) (Fig. 13).

### Over-expression of APETALA2 gene affects the expression of LsGLO genes

**Figure 14:**
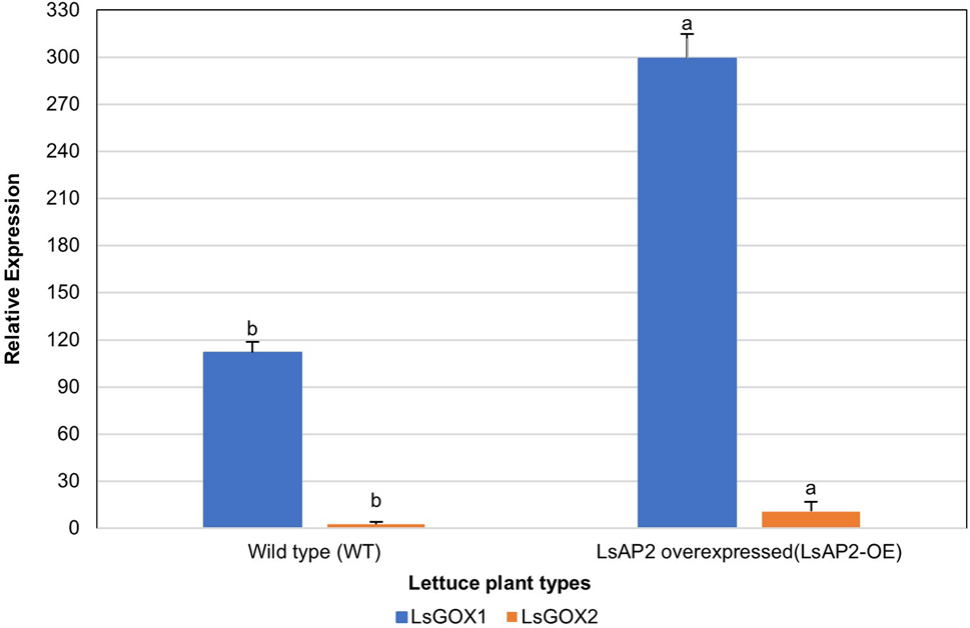
Expression profiling of *LsGLOs* in wild type (WT) and LsAP2 overexpressed (LsAP2-OE) lettuce S39 cultivar. Different letters indicate significant differences determined by one-way ANOVA with Tukey HSD test (*p* < 0.05) and error bars represent standard error in biological replicates. *LsGLO* genes are represented by different colours as shown in legends.

Leaf expression profiling of one-month old wild type (WT) and LsAP2 over-expressed (LsAP2-OE) lettuce plants revealed significant expression (*p* < 0.05) of only two *LsGLO* genes, *LsGOX1* and *LsGOX2*. The expression of both *LsGOX1* and *LsGOX2* genes was high in LsAP2 over-expressed lettuce group compared to wild lettuce (Fig. 14). A significant increase in expression was showed by *LsGOX1*, whereas, *LsGOX2* showed very minor increase in expression in LsAP2 over-expressed lettuce group (Fig. 14).

### Light regulated expression of LsGLOs

RNA sequencing was performed on 24 days old lettuce leaves grown under treatments of varying light intensities (low, medium, and high light intensity). The data revealed that not a single *LsGLO* gene was targeted by light-responsive circular RNAs (circRNAs).

### Expression of LsGLO genes in downy mildew-infected lettuce

**Figure 15:**
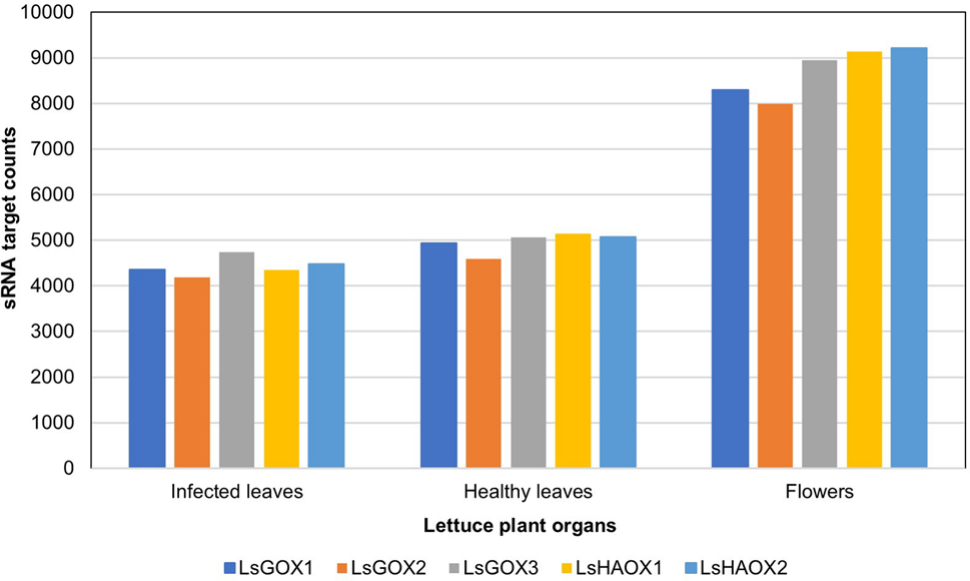
Extent of sRNAs targeting *LsGLO* genes in flowers, healthy lettuce leaves and lettuce leaves infected with *Bremia lactucae*. Different colours represent *LsGLO* genes as shown in legends.

Small RNAs (sRNA) derived from lettuce flowers and healthy and infected (*Bremia lactucae*) lettuce leaves targeted all *LsGLO* genes in both healthy and infected lettuce leaves (Fig. 15). Number of sRNA targets were transcribed as expression of *LsGLO* genes. Graph showed that *LsGLO* genes showed higher expression in heathy leaves than infected lettuce leaves, however, *LsGLOs* showed highest expression in flowers (Fig. 15). *LsHAOX1* showed similar level of expression as *LsGOX3* in healthy leaves. However, expression of *LsGOX1* was lower than *LsGOX3* in infected leaves. *LsHAOX1* and *LsHAOX2* showed similar level of expression in healthy leaves and flowers as *LsGOX3*, however, expression of these genes was lower than *LsGOX3* in infected leaves (Fig. 15).

### Putative miRNA targets in *Lactuca sativa*

Plant MicroRNA Encyclopedia database was used to retrieve the miRNA sequences, and through an online tool, psRNATarget, miRNAs were identified that potentially target lettuce (*Lactuca sativa v8*) *GLO* genes. As a result, six miRNAs were found targeting all five *LsGLO* genes. These miRNAs were single-stranded nucleotides with 20 to 21 nucleic acids (Table 6). The number of miRNAs targeting lettuce *GLO* genes were one miRNA per *LsGLO* genes, and only one miRNA, Lsa-miR414, targeted two *LsGLO* genes. *Lsa-miR168a and Lsa-miRN1713 targeted LsGOX1*. *LsGOX2* and *LsGOX3* were targeted by only one miRNA, Lsa-miR2109 and Lsa-miRN1723, respectively. *LsHAOX*2 was targeted by two miRNAs, Lsa-miR414 and Lsa-miRN1644. *LsHAOX1* was targeted by only one miRNA, Lsa-miR414 (Table 6). This analysis indicated that *LsGOX1* and *LsHAOX2* were the only genes that were targeted by more than one miRNA, and group GOX was targeted the most since this group was targeted by four mature miRNAs, whereas only two miRNAs targeted HAOX group.

**Table 6:** List of miRNAs targeting *GLOs* in *L. sativa*.

miRNA		Target gene	Target		Aligned fragment		Inhibition	Multiplicity
Name	Sequence length		Start	End	miRNA	Target		
Lsa-miR414	20	LsHAOX1	1071	1090	GACGAUGAUGAUGAUGAAGA	GCUUGAUCAUCGCCAUUGUA	Cleavage	1
Lsa-miR414		LsHAOX2						
Lsa-miR168a	21	LsGOX1	1086	1106	UCGCUUGGUGCAGGUCGGGAA	GGCCCGCCCUGCACCACGCUU	Cleavage	1
Lsa-miR2109	20	LsGOX2	688	707	UCAGAGGUGAAGACACUCGU	AAGGGUGUCAUCACUGCUGA	Translation	1
Lsa-miRN1644	20	LsHAOX2	286	305	CUUGUUCUUGUUGUUGUUGU	GCAAGAGCAGCGGGAGCAUG	Cleavage	1
Lsa-miRN1713	20	LsGOX1	293	312	AUUCAUGAUUGAUAGAGCUG	CAGCUGGAACAAUCAUGACU	Cleavage	1
Lsa-miRN1723	20	LsGOX3	928	947	CUCAAUGGGAAAAUAAAAUG	CCUGUCAUUUUCUCAUUGGC	Cleavage	1

**Figure 16:**
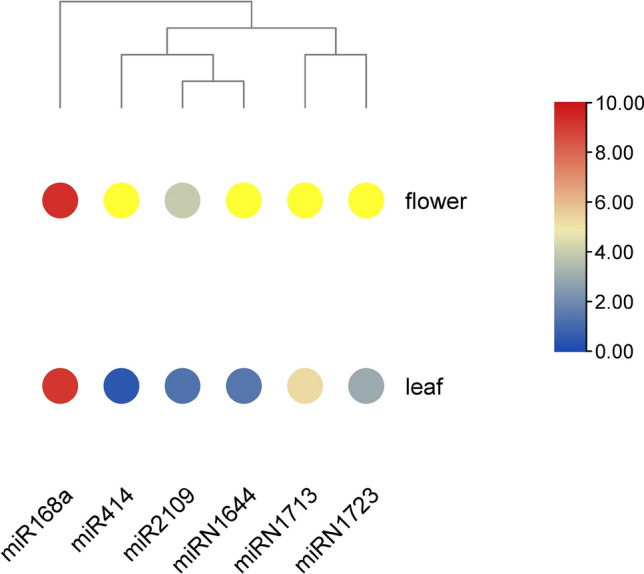
Expression of *LsGLOs* targeting miRNAs in leaf and flower of mature lettuce plants. The expression values are represented as log_2_ fold-change. Color gradient represents the expression levels of miRNAs with blue indicating the lowest, red indicating the highest, and yellow indicating the absence of expression in lettuce.

The expression of these miRNAs in leaf and flower was also studied, which revealed that miR168a showed the highest expression in both leaf and flower and only miR168a and miR2109 showed expression in both leaf and flower, however, the expression showed by miR2109 was too low in comparison with miR168a (Fig. 16). Apart from miR168a and miR2109, no other miRNA was expressed in the flower. miRN1713, miRN1723, and miRN1644 showed expression in the leaf to some extent, but it was very low compared to miR168a (Fig. 16).

## Discussion

This study identified five GLO family genes in lettuce (Table 1). Three domains, namely FMN-dependent dehydrogenase (FMN-dh), long chain alpha-hydroxy acid oxidase (alpha_hydroxyacid_oxid_FMN), and L-lactate dehydrogenase (LldD) were found to be 100% conserved in all lettuce *GLO* genes along with various binding and catalytic sites (Fig. 1, Supplementary material Table S1). In-silico analysis predicted the absence of nuclear localization signals (NLS) in lettuce *GLO* proteins and sub-cellular localization signals confirmed the presence of abundant cytosolic signals in *LsGOX1-3* and peroxisomal signals in *LsHAOX1-2* (Fig. 2, Supplementary material Table S2).

Phylogenetic grouping of *LsGLO* genes was based on the grouping of *Arabidopsis GLO* genes, and the grouping was confirmed by predicted sub-cellular localization signals, since, cytosolic signals were dominant in GOX. Peroxisomal signals were dominant in HAOX group (Fig. 3). In GOX group, *GOX1* and *GOX2* genes of lettuce and *Arabidopsis* shared the same clade. Protein–protein analysis revealed that *LsGOX1* and *LsGOX2* showed maximum percentage homology with *AtGOX1* (Table 2). *LsGOX3* was present in an entirely different clade from *StGOX1* and *SlGOX2,* and the same pattern was observed for *AtGOX3,* which shared the clade with *StGOX2* and *SlGOX3*. *LsGOX3* expressed maximum percentage homology with *AtGOX3*. In HAOX group, *HAOX1* and *HAOX2* genes of lettuce and *Arabidopsis* were present in different clades. *LsHAOX1* and *LsHAOX2* showed the highest homology with *AtHAOX1* (Table 2). The presence of *HAOX* genes of *L. sativa* and *Arabidopsis* far away from each other in comparison with *GOX* genes of these species suggests that *HAOX* genes are evolutionarily more distant as compared to *GOX* genes (Fig. 3). Proteins present in the same clade usually exhibit similar structure and functions. In *Arabidopsis*, only *AtGOX1* and *AtGOX2* genes are involved in the photorespiratory pathway, whereas, *AtGOX3*, *AtHAOX1*, and *AtHAOX2* genes play a role in non-photosynthetic functions^75,76^. The high protein homology of lettuce and *Arabidopsis GLO* genes suggested that *LsGLO* genes might perform in the same pattern (Table 2).

The knowledge about the presence and position of exons and introns in a gene can be used for understanding the gene’s evolutionary relationship with other genes or organisms^71,77^. Analysis revealed that all five *LsGLO* genes contain introns on the upstream end of the *GLO* domain, and similar exon–intron structure was shared among all lettuce *GLO* genes in the same group (Fig. 4, Supplementary material Table S4). Similar exon–intron association noticed in *S. oleracea*, *S. lycopersicum*, *S. tuberosum,* and *Arabidopsis* suggests that these structures remain preserved during the evolutionary process and might protect gene integrity (Fig. 4).

Assessment of conserved motifs in *LsGLO* genes identified twelve motifs conserved in all LsGLO genes, except motif 15, which was only present in *LsHAOX* genes (Fig. 5, Supplementary material Fig. S2, Supplementary material Table S5). The presence of motifs in the same pattern and position suggests that these motifs might play an essential part in activities specific to *GLO* proteins. The presence of motif 15 only in *LsHAOX* genes and motif 12 only in S*. lycopersicum* and *S. tuberosum HAOX* genes indicates that these motifs are specific to *HAOX* genes and could be one of the controlling factors that distinguish *HAOX* genes from *GOX* genes in these species (Fig. 5).

The location of a gene on the chromosome can assist in the prediction of gene duplication events. Gene duplication event could be called tandem duplication if two or more than two genes are present on the same chromosome, whereas the presence of a gene group on different chromosomes could occur as a result of segmental or whole genome duplication (WGD)^78^. Three *LsGLO* genes were located close to each other on chromosome 5, and syntenic analysis revealed the dominance of segmental duplication (Fig. 6). NCBI protein blast showed 97.02 and 83.97 percent homology of *LsGOX1* with *LsGOX2* and *LsGOX3*, respectively, and 95.43 percent homology of *LsHAOX1* with *LsHAOX2*. High protein homology and the presence of *LsGOX1*, *LsGOX2,* and *LsGOX3* on different chromosomes suggests that these genes might be segmentally duplicated*,* and *LsHAOX1* and *LsHAOX2* might have duplicated through tandem duplication as these genes are present on the same chromosome (Fig. 6). The duplication of *LsGOX3* gene appeared to be dispersed according to synteny analysis, and could mean that this gene might have emerged through replicative transposition from *LsGOX1*. This also explains the position of *LsGOX3* in a different clade than *LsGOX1* and *LsGOX2* in phylogram (Fig. 3). The expansion of a gene family is mainly driven through the phenomenon of gene duplication, and the increase in GLO gene member in higher plants (S. lycopersicum, N. benthamiana, and S. tuberosum) may be mainly due to the duplication of FMN-dependent dehydrogenase (FMN-dh), alpha hydroxyacid oxidase, and L-lactate dehydrogenase domain during evolution of eukaryotic plants^79,80^.

Comparative syntenic maps can be used further to explore the potential evolutionary mechanism of a gene family^81^. Syntenic maps revealed collinear blocks between *LsGOX1*, *AtGOX2*, *SlGOX1*, *StGOX2*, and *StGOX3*, whereas, *LsHAOX2* showed syntenic gene pair with *AtHAOX1*, *SlHAOX1*, and *StHAOX2* (Fig. 7, Table 3), which suggests the origin of these genes from a common ancestor, and explain expansion of *GLO* gene family. The high number of collinear gene pairs between *L. sativa* and *S. lycopersicum*/*S. tuberosum* and the presence of *GLO* genes of these species in the same clades points to their close evolutionary relationship. Syntenic gene pairing of lettuce *GOX* and *HAOX* genes being consistent with *GOX* and *HAOX* genes in respective plant genomes, except in *S. oleracea*, further proves that *LsGOX* genes might be segmentally duplicated *HAOX* genes might have duplicated through tandem duplication. In *S. oleracea*, *LsHAOX2* showed syntenic gene pair with Spov3_chr4.04560 gene, which is not a glycolate oxidase gene, but rather a nuclear pore complex protein Nup107 (NUP107, NUP84) (Table 3). Interestingly, we found that a transcript of *AtGOX1* (AT3G14120.2) is also a nuclear pore complex protein NUP107 which suggests the possibility that *GLO* genes might have evolved from nuclear pore complex proteins, or they might have evolved into nuclear pore complex proteins. Because no *LsGLO* gene or gene isoform is a nuclear pore complex protein, no other *LsGLO* gene showed a syntenic relationship with nuclear pore complex protein, and none of the lettuce *GLO* genes exhibit nuclear localization signals (NLS), the former hypothesis seems more convincing, however, it’s not quite supported by evidence.

The selection pressure on the substitution of amino acids can be understood through the ratio of Ka/Ks (Fig. 8). The ratio of Ka/Ks > 1 suggests the possibility of purifying selection, whereas, Ka/Ks ratio > 1 suggest the likelihood of positive selection^82,83^. Generally, the evaluation of selective pressure provides selective lead for amino acid sequence altered in a protein and is also necessary for interpreting functional residues and protein shifts^84^. The estimated values of Ka/Ks ranged from 0.03 to 0.29, which being less than 1, suggests that lettuce *GLO* genes undergo strong purifying selection pressure and positive selection might have acted on a few sites only during the process of evolution (Fig. 8).

The presence of hormone-responsive, stress and defense responsive *cis*- acting elements suggest that these genes might play important roles in lettuce plant growth and development and might be involved in lettuce plant’s defense mechanisms as well (Fig. 9). Highest number, twelve, of *cis*- acting elements were found in *LsHAOX* genes, and a range of eight to ten *cis*- acting elements were found in *LsGOX* genes (Fig. 9). Light, hormone, and stress-responsive *cis*- acting elements were dominant and defense related *cis*- acting elements were absent in *LsHAOX* genes, whereas, only light and defense related *cis*- acting elements were dominant in *LsGOX* genes. Overall, light responsive *cis*- acting elements were highly dominant, and defense-related *cis*- acting elements were least dominant in lettuce *GLO* genes (Fig. 9, Supplementary material Table S6).

Protein–protein interaction is crucial in understanding the functions of a gene in silico^85^. During cell signaling and metabolism, enzyme activity is known to be regulated through protein–protein interaction^86^. Specificity of the hydrogen peroxide biosynthetic process to only *LsHAOX1-2* genes, and glyoxylate bypass and glycolate oxidase activity being specific to only *LsGOX1-3* genes further confirms the hypothesis that *LsGOX1* and *LsGOX2* play role in photorespiratory pathway and *LsGOX3*, *LsHAOX1*, and *LsHAOX2* genes are involved in non-photosynthetic functions (Table 4). Butanoate metabolism produces signal molecule GABA (g-Aminobutyric acid) that rapidly accumulates in plant tissues in response to biotic and abiotic stress and elevates plant stress tolerance^87^. It is predicted only in *LsGOX1* and *LsGOX3*, which suggests that these genes trigger GABA synthesis by activating butanoate metabolism thus, indirectly enhancing plant defenses in response to plant-animal and plant–microbe interactions (Table 4). Surprisingly, *LsGOX1* and *LsGOX2* did not show any type of interaction with each other. The same thing was observed between *LsHAOX1* and *LsHAOX2* (Fig. 10). The respective gene pairs in *A. thaliana* have a well-established interaction, co-occurrence and co-expression of *AtGOX1/AtGOX2* and co-occurrence, of *AtHAOX1/AtHAOX2*, which implies that these gene pairs in *L. sativa* (*LsGOX1/LsGOX2* and *LsHAOX1/LsHAOX2*) are not regulated or expressed similarly. Glycolate oxidase (GLO) is the first enzyme in glycolate pathway^88,89^, and co-expression of *LsGLO* genes with other members of this pathway (GLYR1, HPR1, and AGT1) emphasizes their strong functional association with *LsGLO* proteins and implies that disruption in this protein will probably result in disruption of the whole glycolate/glyoxylate pathway, photorespiration, and photosynthesis, so the implications would be quite destructive (Fig. 10, Table 5). Co-expression of catalases with *GLO* genes prevent hydrogen peroxide mediated stress in plant due to activity of glycolate oxidases in *A. thaliana*. It is quite surprising that *GLO* genes in *L. sativa* did not interact with catalases despite containing the same number of *GLO* genes as *A. thaliana* and no such stress is reported to date. This suggests that the absence of co-expression interaction between *LsGOX1/LsGOX2* and *LsHAOX1/LsHAOX2* might be directly linked with low hydrogen peroxide mediated stress due to limited activity of glycolate oxidases in lettuce (Fig. 10).

Expression data analysis provides important information to predict the functions of a gene or gene family in an organism and revealed interesting findings. *LsGOX1* and *LsGOX2* genes were up regulated when lettuce plants were grown under low (4 °C) temperature for 24 h as compared to control group (0 h) (Fig. 11). The initial decrease in expression could be the result of shock caused by sudden temperature decrease in plant environment. During short-term chilling stress (4 h), hydrogen peroxide up regulates cyclic electron flow (CEF) and alternative oxidase (AOX) pathways through signal transduction^46^. Alternative oxidase (AOX) optimizes photosynthesis during low temperature, and drought stress^90^. Photorespiration is pivotal for photosynthesis and oxidative stress response was induced, therefore, the expression of the *GLO* genes was increased and coordination among these two processes helps plant to adapt in cold environment. When the stress lasted for longer, i.e., 7 days, the expression of *GLO* genes was decreased due to reduced enzyme activity. The expression of *LsGLO* genes was down regulated in non-treated leaves, melatonin-treated leaves and melatonin-treated roots, and up-regulated in non-treated roots under cadmium stress (Fig. 13). Melatonin is a plant signaling molecule that restricts cadmium (Cd) transport from roots to shoots to protect the photosynthetic apparatus of the plant. Exogenous melatonin stimulates the production of endogenous melatonin. Since melatonin is a ubiquitous antioxidant it aids plants in stress tolerance by enhancing the production of antioxidants and activates the genes involved in redox reactions^91,92^. Movement of glycolate oxidases from other plant parts to roots, increased activity of *LsGLOs* in non-treated roots, and reduced activity in melatonin-treated roots under the same conditions suggests that *LsGLOs* might also play a role in increasing stress tolerance of lettuce through an increase in redox reactions and H_2_O_2_-mediated signal transduction under heavy metal (Cd) stress. These results support the role of *LsGLO* genes in lettuce tolerance to abiotic stresses.

The expression of *LsGOX1* was up regulated during the vegetative, bolting, and inflorescence stage in bolting-sensitive line S39 as compared to bolting-resistant line S24 (Fig. 12). High temperature promotes bolting and also enhances photorespiration which explains high expression of *LsGOX1* and suggests that *LsGOX1* might be a key player in plant developmental processes such as inflorescence and flower initiation. The down regulation of *LsGOX1* at a bolting stage in bolting-resistant line S24 could be due to changes in lettuce plant gene expression to resist bolting (Fig. 12). Expression pattern of *LsGOX1* and *LsGOX2* in *APETALA2* over-expressed lettuce plants was significantly higher as compared to wild type lettuce (Fig. 14). This suggests that *LsGOX1* might play role in leaf development. The number of sRNAs targeting *LsGLO* genes in flowers, healthy and infected lettuce leaves can be interpreted as the expression level of *LsGLO* genes in these organs. sRNA targets demonstrated that *GLO* genes were down regulated in infected (downy mildew) lettuce leaves (Fig. 15). Lettuce plant, when exposed to *Bremia lactucae* activates SAR (Systemic acquired resistance) signaling in which known compound such as abscisic acid, methyl-jasmonate, ethylene, sodium-salicylate are involved. Sodium-salicylate is a salicylic acid derivative and salicylic derivatives have been found to inhibit glycolate oxidases through binding to the substrate site, decreasing *GLO* gene expression^93,94^. Significantly high expression level of *LsGLO* genes in flowers as compared to healthy lettuce leaves suggests the involvement of these genes in various processes and functions related to flowering. These results suggest that *LsGOX1* might play vital role in flowering initiation and leaf and flower developmental processes.

*LsGLO* genes are not regulated by light intensity since no *GLO* gene was targeted by light-responsive circular RNAs (circRNAs). Six miRNAs were found to target the *GLO* genes in mature lettuce plants, but only miR168a was significantly expressed in mature lettuce flowers and leaves that specifically targeted *LsGOX1* (Fig. 16, Table 6). According to previous studies, MIR168 has been most commonly detected as stress inducible gene and MIR168a is abundant in rice^95,96^. 21 nucleotide MIR168 showed effective response to fluctuations in plant environment^97^. MIR168 regulates the function of miRNAs, since it regulates the expression of *AGO1* (Argonaute RISC Component 1) and in *Arabidopsis*, and it is involved in post-transcriptional gene silencing by maintaining *AGO1* homeostasis^98–101^. High expression of miR168a in leaf and flower might result ofresult from the post-transcriptional gene silencing of *LsGOX1* to regulate its expression. MIR168a regulates flowering time and is involved in plant hormone signal transduction and plant-pathogen interaction in rice^95,96^. This supports the previously stated roles of *LsGOX1* during inflorescence and flowering stage and in various stresses suggesting that *LsGOX1* might play role in regulating flowering, plant signaling and interaction with pathogens.

The results of all these expression analyses suggest that *LsGOX1* and *LsGOX2* might play role in plant resistance mechanism by regulating ROS-mediated signaling during various environmental stresses, *LsGOX1* seems to contribute in various stages of plant development through oxidative photosynthetic carbon pathway and both of these functions are also performed by *AtGOX1* (Table 6). miR168a targeting *LsGOX1* might regulate gene expression of *LsGOX1* through AGO1-mediated negative feedback and avoid abnormality in plant functioning or any developmental defects.

In *Arabidopsis*, the combined expression of *GOX1* and *GOX2* was reported to be higher than *GOX3* in leaves. Lower in roots under normal conditions@@^102,103^, and the same results were observed in lettuce (Figs. 11, 13). However, Arabidopsis leaves infected with Botrytis cinerea showed reduction in combined expression of GOX1 and GOX2. It became comparable to the expression of GOX3 and same pattern in LsGOX genes was observed in B. lactucae-infected lettuce leaves (Fig. 16). In Arabidopsis, AtGOX1 and AtGOX2 genes were highly expressed in green organs, and these genes are co-expressed with other AtGLO genes@@^21,104^. The same pattern was observed in lettuce *GLO* genes. In conclusion, lettuce glycolate oxidase (*GLO*) might be a constitutive gene, as it is expressed in all organs. The significance of its expression include uninterrupted photorespiration essential for photosynthesis in C3 plants like lettuce, plant signaling in response to plant–microbe interactions, developmental role in leaf and flower, and tolerance to abiotic stresses.

## Material and methods

It has been confirmed that the experimental data collection complied with relevant institutional, national, and international guidelines and legislation with appropriate permissions from authorities of the Department of Horticulture, Faculty of Agricultural Sciences, University of the Punjab, Lahore, Pakistan.

### Database search and retrieval of sequence

The GLO protein amino acid sequence was retrieved from *S. oleracea* (Accession no *SoGLO1*/Spo19861) through SpinachBase database^105,106^. 341 AA sequence of FMN-dependent dehydrogenase domain was retrieved from *SoGLO1* (Accession no Spo19861) through Pfam database. The peptide sequence of *SoGLO1*/Spo19861 was used to identify *GLO* protein encoding genes in lettuce (*Lactuca sativa cv. Salinas/L. sativa v8*) proteome database at Phytozome v13 using BLAST-P 2.6.0 + (Protein- basic local alignment search tool) program^107,108^. Furthermore, the protein sequences of the reported *Arabidopsis GLO* family genes were retrieved from TAIR at Phytozome v13, which consisted of three *AtGOX* (AT3G14420, AT3G14415 and AT4G18360), and two *AtHAOX* (AT3G14130 and AT3G14150) genes. These sequences were then used to BLAST-P (Protein- basic local alignment search tool) search in *Lactuca sativa v8* (*Lactuca sativa cv. Salinas*) proteome database at Phytozome v13 to reconfirm the retrieved *GLO* proteins in lettuce.

### Identification of conserved domains in LsGLO genes

The retrieved amino acid sequences were subjected to NCBI CDD (Conserved Domain Database) with the default parameters^109,110^. The proteins lacking FMN-dependent dehydrogenase (PF01070) conserved domain were removed.

### Determining physio-chemical properties of LsGLO proteins

The length of proteins (amino acid), weight of protein molecules, and theoretical isoelectric point of *LsGLO* proteins were predicted through an online tool, ProtParam^111^. The information regarding gene identity, position on chromosomes and gene and protein sequences were retrieved from Phytozome. The renaming of *LsGLO* genes was done based on the order of their physical position in lettuce genome database.

### Prediction of nuclear and sub-cellular localization signals

The nuclear localization signals (NLS) in lettuce *GLO* proteins were predicted through an online server, nuclear localization signals database (NLSdb)^112–114^. Subcellular localization of *LsGLO* proteins was predicted using an online tool WoLF PSORT^115^.

### Phylogenetic analysis

The amino acid sequence of *GLO* proteins in *L. sativa*, *S. oleracea*, *S. lycopersicum*, *S. tuberosum*, and *A. thaliana* along with HPR, GLYR, AGT, UAH, MLS, and CAT protein sequences of *L. sativa* and *A. thaliana* were aligned using Clustal W version 2.1^116,117^. Neighbor joining method (NJ) in MEGA X v10.2.4 program created the phylogenetic tree with bootstrap set at 1000 replications and other default parameters^118,119^. HPR, GLYR, AGT, UAH, MLS, and CAT proteins were used as outgroup.

### Gene structure analysis

Genomic and coding sequence of *LsGLO* genes were retrieved from Phytozome database and were used to draw the gene structure through Gene Structure Display Server (GSDS v2.0)^120,121^.

### Recognition of conserved motifs

Multiple EM for Motif Elicitation (MEME) program was used to analyze motifs in the retrieved *LsGLO* protein sequences with maximum number of motifs set as 15, and minimum 6 and maximum 50 width of motif was set as default values along with other factors^122,123^. Visual representation of the motifs was created using the Gene Structure View (Advanced) software in TBtools^124^.

### Identification of LsGLO gene orthologs in arabidopsis

Protein homologs of *L. sativa* GLO genes were identified in *A. thaliana* through protein–protein BLAST (blastp) on NCBI, a web-interface^125,126^. The analysis was conducted using protein sequences of *LsGLO* genes against the whole genome of *A. thaliana* (taxonomy id: 3702) with all parameters set as default.

### Chromosomal location and synteny analysis

The information regarding chromosome length and location of gene on chromosome was extracted from the lettuce genome database, and gene location was visualized through TBtools. Gene pairs of lettuce *GLO* genes were created through TBtools, and synteny analysis was conducted using the Advanced Circos program in TBtools. The genome and gff3 files of *L. sativa*, *A. thaliana*, *S.oleraceae*, *S. lycopersicum* and *S. tuberosum* were retrieved from Phytozome database to display the syntenic blocks of *L. sativa GLO* genes in respective genomes. The syntenic maps were constructed by using the One Step MCScanX and Dual Systeny Plot in TBtools comparative genomics program with default parameters^124,127,128^.

### Analysis of gene duplication event

The lettuce GLO gene family duplication event was studied using Ks and Ka values. The number of non-synonymous (Ka) and synonymous (Ks) substitution rates, and Ka/Ks ratios were calculated using the simple Ka/Ks calculator through TBTools Software^124^. The parameters were set as described in the software package manual. The Ka/Ks ratios were used to predict the rates of molecular evolution of each paralogous gene pair^5^. Generally, a Ka/Ks ratio greater than one indicates positive selection, a ratio close to 1 indicates neutral selection, whereas, a ratio less than 1 indicates the probability of purifying selection which leads to limited functional divergence of the duplicated genes^5^.

### Analysis of cis-acting elements

A sequence of 1000 bps upstream was retrieved from the initiation codon of putative LsGLO genes to analyze the promoter region. PlantCare database was then used to predict the cis-regulatory elements in these sequences^129–132^. The number and position of the predicted cis-regulatory elements were visualized using a heat map and Basic Biosequence view program in TBtools^133–135^.

### Analysis of protein–protein interaction network

Interactions among the lettuce *GLO* proteins were studied by constructing protein interaction networks using the Search tool in STRING database v11.5 with a medium confidence score of 0.400 as the default value, along with other settings. Functional associations in interacting networks consisted of biological processes, molecular functions, KEGG pathways, and local network clusters created by STRING database v11.5^136,137^.

### Transcriptome analysis

To study the external stimuli-specific expression profile of *LsGLOs* in various organs, we analyzed previously generated RNA-seq data of lettuce (*L. sativa*) plant under several biotic, abiotic, and developmental stresses from NCBI GEO (Gene Expression Omnibus). These stresses include low temperature, light, heavy metal (Cd), bolting, over-expression of *APETELA2,* and lettuce downy mildew (*Bremia lactucae*) infection. Statistical analysis of the gene expression data was conducted through Statistix v8.1 (Analytical software 2005).

### Effect of low temperature (4 °C) stress on LsGLO genes

Lettuce (*Salinas*) plants were grown at 20 °C in a growth chamber, and 18-day old plants were exposed to low temperatures (4 °C) for different time intervals, i.e., 4 h, 24 h, and 7 days^5^. ‘0 h’ sample represents controlled group lettuce plants grown at 20 °C. Three replicates of lettuce plant were taken for each time interval, so the total number of sample plants taken was 12. RNA sequencing was performed in above-ground lettuce plant tissues, and expression data was calculated in log2FPKM. Statistical analysis was done using ANOVA, genes showing *p* < 0.05 were considered statistically significant and Tukey HSD test determined the significant differences in expression.

### Lettuce plant under bolting stress

An experiment was conducted to understand the underlying processes in inflorescence development and the putative role of *LsFT* (FLOWERING LOCUS T) in the regulation of bolting in lettuce. Two leafy lettuce lines, bolting-sensitive line S39, and bolting-resistant line S24 of lettuce (*L. sativa*) were cultivated in a growth chamber at 25 °C day and 15 °C night temperature. Expression profiling of shoot apical meristem (SAM) cells was carried out at four critical developmental stages in lettuce i.e., vegetative stage from 0 to 35 days after planting (DAP), bolting stage from 35 to 75 DAP, inflorescence stage from 75 to 95 DAP, and flowering stage from 95 to 125 DAP. Two replicates of shoot apical meristem (SAM) cells were taken from each developmental stage of both lettuce lines, so 16 replicates were created for experiment^48^. Expression values were calculated in log2FPKM. Significance in *LsGLO* expression was determined by p-value, genes showing *p*-value < 0.05 were considered differentially expressed.

### Leaves and roots of *L. sativa* under cadmium (Cd) stress

A study was conducted to understand the relationship between exogenous melatonin treatment and relative resistance shown by Italian Lettuce (*L. sativa var. ramose Hort.*) against cadmium (Cd) stress environment^138^. The seeds were germinated in a growth chamber and after 20 days, the seedlings were divided into two groups based on the treatments applied i.e., seedlings cultivated with a nutrient solution containing 1 μmol/L (Sigma-Aldrich, USA) melatonin, and seedlings cultivated with a nutrient solution without melatonin (control group). Then after two days, both groups were treated with 50 μmol/L cadmium chloride (CdCl_2_) to create Cd stress environment. The leaves and roots of seedlings in two groups were sampled at 0-, 1-, and 5-days interval and three replicates were taken for each treatment. Expression levels were calculated in log2FPKM. Significant expression was determined through multiple comparisons of means were performed using Tukey HSD test at significance level of 0.05.

### Over-expression of APETALA2 in lettuce leaves

Wild type (WT) and LsAP2 overexpressed (LsAP2-OE) S39 cultivar of leaf lettuce (*Lactuca sativa L.*) plants were cultivated in a growth chamber at 16/8 h light/dark and 25/18 °C day/night temperature. Three replicates were taken from each treatment, and RNA-seq experiment was conducted on leaves of each sample after one month^139^. Expression data was calculated in log2FPKM. Significance in gene expression was determined by one-way ANOVA (*p*-value < 0.05) and Tukey HSD test.

### Lettuce plant under light stress of various intensities

RNA sequencing (RNA-Seq) was performed on 24 days old lettuce leaves grown in 3 different light intensity treatments, low light (Las_WL, 60 ± 2 µmol m^−2^ s^−1^), medium light (Las_ML, 175 ± 2 µmol m^−2^ s^−1^), and high light intensity (Las_SL, 340 ± 2 µmol m^−2^ s^−1^) in a growth chamber^57^. Expression data was calculated in log2FPKM. Differentially expressed genes were selected based on log_2_(fold-change) > 1, and statistical significance (*p* < 0.05) was determined by using ANOVA.

### Expression profiling of LsGLO genes in various organs through sRNAs targets

An experiment was designed in which lettuce leaves infected with fungus *Bremia lactucae* strain CA-III were harvested 7 days after inoculation and small RNA libraries were derived from flowers, heathy leaves of lettuce (*Lactuca sativa cv. ‘Salinas’*) and infected (*Bremia lactucae*) lettuce leaves (GEO accession: GSE28322). Analysis was conducted by uploading the sequence of small RNAs and CDS sequences of all lettuce *GLO* genes on an online tool, psRNATarget with default parameters^64,140^. The expression pattern of *LsGLO* genes was visualized through bar graph.

### Analysis of putative microRNAs targeting LsGLO genes

The lettuce plant's micro-RNA (miRNA) dataset was retrieved from PmiREN (Plant miRNA Encyclopedia)^141^. To find out the miRNAs which targets the lettuce *GLO* genes, the CDS sequences of all *GLO* genes of lettuce were searched against the complementary sequences of miRNAs with the help of psRNATarget with default parameters^64,140^. Expression levels of miRNA in leaves and flowers were presented in log_2_ change. Heatmap illustrator in TBtools was used to display expression patterns of miRNAs in lettuce plants with hierarchical clustering^124,133^.

### Supplementary Information

Supplementary Information.

## Data Availability

All the data generated or produced during the study has been given in the manuscript and its related supplementary file. Gene expression data studies in this paper were downloaded from NCBI GEO (Gene Expression Omnibus) (Series Accession: GSE134012, GSE108260, GSE143675, GSE168886, GSE148578, GSE28322).
