# Supplementary material for: Identification and characterization of Glycolate oxidase gene family in garden lettuce (Lactuca sativa cv. ‘Salinas’) and its response under various biotic, abiotic, and developmental stresses
Source: Sci Rep. 2023 Nov 11;13:19686. doi: 10.1038/s41598-023-47180-y (PMC10640638; doi:10.1038/s41598-023-47180-y)
Supplement: Supplementary file 1 — Supplementary Information. [file 41598_2023_47180_MOESM1_ESM.docx]

**Supplementary material figures and tables**

**Mariyam^1^, Muhammad Shafiq^*1^, Saleha Sadiq^2^, Qurban Ali^*3^, Muhammad Saleem Haider^4^, Umer Habib^5^, Daoud Ali^5^, Muhammad Adnan Shahid^6^**

^1^Department of Horticulture, University of the Punjab, Lahore, Pakistan ([mariyamirfan36@gmail.com](mailto:mariyamirfan36@gmail.com), [shafiq.iags@pu.edu.pk](mailto:shafiq.iags@pu.edu.pk))

^2^Department of Biotechnology, The Islamia University of Bahawalpur, Bahawalpur, Pakistan, ([salehasadiqape@gmail.com](mailto:salehasadiqape@gmail.com))

^3^Department of Plant Breeding and Genetics, University of the Punjab, Lahore 54590, Pakistan ([saim1692@gmail.com](mailto:saim1692@gmail.com))

^4^Department of Plant Pathology, University of the Punjab, Lahore, Pakistan ([haider65us@yahoo.com](mailto:haider65us@yahoo.com))

^5^Department of Horticulture, PMAS Arid Agriculture University, Murree Road, Rawalpindi, Pakistan ([umer@uaar.edu.pk](mailto:umer@uaar.edu.pk))

^5^Department of Zoology College of Science King Saud University, PO Box 2455 Riyadh 11451, Saudi Arabia ([Aalidaoud@ksu.edu.sa](mailto:Aalidaoud@ksu.edu.sa))

^6^Horticultural Sciences Department, University of Florida/IFAS, North Florida Research and Education Center, Quincy, FL 32351, USA ([mshahid@ufl.edu](mailto:mshahid@ufl.edu))

*Corresponding Authors Email; [shafiq.iags@pu.edu.pk](mailto:shafiq.iags@pu.edu.pk), [saim1692@gmail.com](mailto:saim1692@gmail.com)

**Supplementary material Table S1 Brief information of the domains found in *L. sativa* glycolate oxidase proteins**

| **Domain** | **Detail** |
| --- | --- |
| FMN_dh | FMN-dependent dehydrogenase |
| alpha_hydroxyacid_oxid_FMN | Family of homologous FMN-dependent alpha-hydroxyacid oxidizing enzymes. This family occurs in both prokaryotes and eukaryotes |
| LldD | L-lactate dehydrogenase |
| L_lactate_LldD | FMN-dependent L-lactate dehydrogenase LldD |
| TIM superfamily | TIM-like beta/alpha barrel domains; A large family of domains similar to triose phosphate isomerase (TIM) which, in general, share an eight beta/alpha closed barrel structure |
| PLN02493 | Probable peroxisomal (S)-2-hydroxy-acid oxidase |
| PLN02535 | Glycolate oxidase |

**Supplementary material Table S2 Nuclear localization signals (NLS) and sub-cellular localization prediction of lettuce glycolate oxidase proteins**

| **Group** | **GLO genes** | **Chromosome no** | | **NLS Signal** | **Sub-cellular location** | | | | | |
| --- | --- | --- | --- | --- | --- | --- | --- | --- | --- | --- |
|  |  |  |  |  | **Cyto** | **Pero** | **Chlo** | **Mito** | **Cysk_nucl** | **Plas** |
| **GOX** | LsGOX1 | Lg 9 | | N/A | 05 | 04 | 02 | 02 | 01 | 0 |
|  | LsGOX2 | | Lg 5 | N/A | 07 | 02 | 02 | 02 | 0 | 01 |
|  | LsGOX3 | Lg 4 | | N/A | 09 | 03 | 01 | 01 | 0 | 0 |
| **HAOX** | LsHAOX1 | Lg 5 | | N/A | 02 | 12 | 0 | 0 | 0 | 0 |
|  | LsHAOX2 | Lg 5 | | N/A | 01 | 13 | 0 | 0 | 0 | 0 |

Note: Cyto: cytosol; Pero: peroxisomes; Chlo: chloroplast; Mito: mitochondria; Plas: plasma membrane; Cysk_nucl: cytoskeleton and nucleus

**Supplementary material Table** S**3 Accession numbers of non-redundant glycolate oxidase genes found in *S. oleracea*, *S. lycopersicum*, and *S. tuberosum***

| **GLO Genes** | **Accession No.** | **GLO Genes** | **Accession No.** |
| --- | --- | --- | --- |
| **SlGLO1 (SlGOX1)** | Solyc07g056540 | **StGLO1 (StGOX1)** | Soltu.DM.08G007550 |
| **SlGLO2 (SlGOX2)** | Solyc08g048250 | **StGLO2 (StGOX2)** | Soltu.DM.10G003260 |
| **SlGLO3 (SlGOX3)** | Solyc10g007600 | **StGLO3 (StGOX3)** | Soltu.DM.07G022590 |
| **SlGLO4 (SlHAOX1)** | Solyc03g122130 | **StGLO4 (StHAOX1)** | Soltu.DM.03G036940 |
| **SlGLO5 (SlHAOX2)** | Solyc03g122140 | **StGLO5 (StHAOX2)** | Soltu.DM.03G036930 |
| **SlGLO6 (SlHAOX3)** | Solyc03g122170 | **StGLO6 (StHAOX3)** | Soltu.DM.03G036950 |
| **SoGLO1 (SoGOX1)** | Spo19861 | **StGLO7 (StHAOX4)** | Soltu.DM.03G036960 |
| **SoGLO4 (SoGOX2)** | Spo21282 | **SoGLO2 (SoHAOX1)** | Spo21903 |
| **SoGLO5 (SoGOX3)** | Spo20781 | **SoGLO3 (SoHAOX2)** | Spo10076 |

**Supplementary material Table S4 In silico predicted number of introns and exons in lettuce glycolate oxidase genes**

| **Groups** | **GLO genes** | **Accession no.** | **Intron no.** | **Exon no.** |
| --- | --- | --- | --- | --- |
| **GOX** | LsGOX1 | Lsat_1_v5_gn_9_103440 | 09 | 10 |
|  | LsGOX2 | Lsat_1_v5_gn_5_120941 | 08 | 09 |
|  | LsGOX3 | Lsat_1_v5_gn_4_29481 | 09 | 10 |
| **HAOX** | LsHAOX1 | Lsat_1_v5_gn_5_136840 | 10 | 11 |
|  | LsHAOX2 | Lsat_1_v5_gn_5_136860 | 10 | 11 |

**Supplementary material Table S5 Detailed information of the motifs found in glycolate oxidase proteins**

| **ID** | **Motif** | **Domain** | **Length**  **(AA)** | **Sites** |
| --- | --- | --- | --- | --- |
| **1** | GIIVSNHGARQLDYVPATISALEEVVKAVQGRIPVFLDGGVRRGTDVFKA | [IMPDH](https://www.genome.jp/dbget-bin/www_bget?pf:IMPDH) | 50 | 28 |
| **2** | ENRNAFSRILFRPRILIDVSKIDMSTTILGFKISMPIMIAPTAMQKMAHP | FMN-dh | 50 | 28 |
| **3** | SGLASYVAGQIDRSLSWKDVAWLQSITKLPILVKGVLTAED | FMN-dh | 41 | 28 |
| **4** | RBVVAQLVRRAEKAGFKAIALTVDTPRLGRREADIKNRFILPP | FMN-dh | 43 | 27 |
| **5** | VFIGRPVIYSLAAKGEAGVKKVJZMLKDEFELTMALSGCRSLKEITRNHV | FMN-dh | 50 | 26 |
| **6** | TARAASAAGTIMTLSSWATSSVEEVASTGPGVRFFQLYVYK | FMN-dh | 41 | 24 |
| **7** | NVNEYZEJAKQKLPKMYYDYYASGAEDQW | FMN-dh | 29 | 23 |
| **8** | LKNFEGLDLGKVDKA | - | 15 | 26 |
| **9** | LALGAS | - | 06 | 27 |
| **10** | ARKAVZAG | - | 08 | 26 |
| **11** | VTEWDTPRARP | - | 11 | 17 |
| **12** | KTEKERFLCRM | - | 11 | 07 |
| **13** | MYYDYYAGGAEDQWT | - | 15 | 04 |
| **14** | CSVYMVYKD | - | 09 | 04 |
| **15** | MAGEPV | - | 06 | 06 |

**Supplementary material Table** S**6 Brief description of the *cis*- regulatory elements present in *LsGOX* proteins**

| **Site Name** | **Organism** | **Sequence** | **Function** |
| --- | --- | --- | --- |
| **AE-box** | *Arabidopsis thaliana* | AGAAACAA | part of a module for light response |
| **Box 4** | *Petroselinum crispum* | ATTAAT | part of a conserved DNA module involved in light responsiveness |
| **GATA-motif** | *Pisum sativum* | GATAGGG | part of a light responsive element |
|  | *Solanum tuberosum* | AAGGATAAGG |  |
| **I-box** | *Triticum aestivum* | AGATAAGG | part of a light responsive element |
|  | *Helianthus annuus* | ATGATAAGGTC |  |
|  | *Flaveria trinervia* | CCATATCCAAT |  |
|  | *Arabidopsis thaliana* | CCTTATCCT |  |
| **GT1-motif** | *Avena sativa* | GGTTAAT | light responsive element |
| **MRE** | *Petroselinum crispum* | AACCTAA | MYB binding site involved in light responsiveness |
| **Sp1** | *Oryza sativa* | GGGCGG | light responsive element |
| **G-Box** | *Pisum sativum* | CACGTT | cis-acting regulatory element involved in light responsiveness |
| **TCT-motif** | *Arabidopsis thaliana* | TCTTAC | part of a light responsive element |
| **CGTCA-motif** | *Hordeum vulgare* | CGTCA | cis-acting regulatory element involved in the MeJA-responsiveness |
| **TCA-element** | *Nicotiana tabacum* | CCATCTTTTT | cis-acting element involved in salicylic acid responsiveness |
| **TGACG-motif** | *Hordeum vulgare* | TGACG | cis-acting regulatory element involved in the MeJA-responsiveness |
| **ARE** | *Zea mays* | AAACCA | cis-acting regulatory element essential for the anaerobic induction |
| **GC-motif** | *Zea mays* | CCCCCG | enhancer-like element involved in anoxic specific inducibility |
| **MBS** | *Arabidopsis thaliana* | CAACTG | MYB binding site involved in drought-inducibility |
| **TC-rich repeats** | *Nicotiana tabacum* | GTTTTCTTAC | cis-acting element involved in defense and stress responsiveness |
| **P-box** | *Oryza sativa* | CCTTTTG | gibberellin-responsive element |
| **ABRE** | *Hordeum vulgare* | CGTACGTGCA | cis-acting element involved in the abscisic acid responsiveness |
| **TATC-box** | *Oryza sativa* | TATCCCA | cis-acting element involved in gibberellin-responsiveness |
| **TGA-element** | *Brassica oleracea* | AACGAC | auxin-responsive element |
| **AT-rich element** | *Glycine max* | ATAGAAATCAA | binding site of AT-rich DNA binding protein (ATBP-1) |
| **CCAAT-box** | *Hordeum vulgare* | CAACGG | MYBHv1 binding site |
| **TATA-box** | *Arabidopsis thaliana* | TATATA | core promoter element around -30 of transcription start |
|  | *Brassica napus* | ATATAT |  |
|  | *Helianthus annuus* | TATACA |  |
|  | *Oryza sativa* | TACAAAA |  |
|  | *Lycopersicon esculentum* | TATATAAAG |  |
| **CAAT-box** | *Arabidopsis thaliana* | CCAAT | common cis-acting element in promoter and enhancer regions |
|  | *Pisum sativum* | CAAAT |  |
|  | *Nicotiana glutinosa* | CAAT |  |
| **GCN4_motif** | *Oryza sativa* | TGAGTCA | cis-regulatory element involved in endosperm expression |
| **CAT-box** | *Arabidopsis thaliana* | GCCACT | cis-acting regulatory element related to meristem expression |
| **O2-site** | *Zea mays* | GATGACATGG | cis-acting regulatory element involved in zein metabolism regulation |
| **MBSI** | *Petunia hybrida* | TTTTTACGGTTA | MYB binding site involved in flavonoid biosynthetic genes regulation |

**
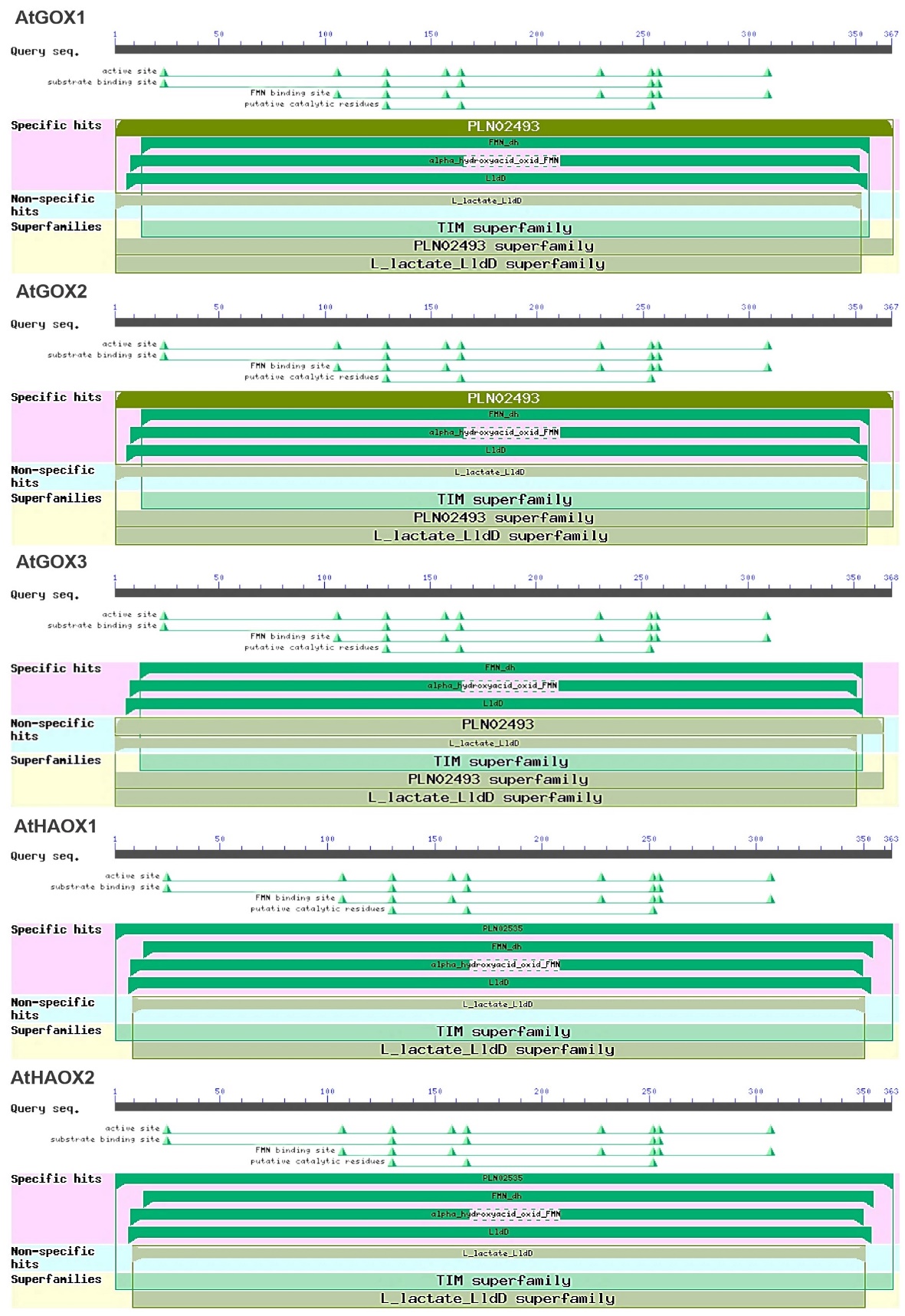
**

**Supplementary material Figure S1 Identification of conserved domains in Arabidopsis through NCBI CDD using full length sequence of GLO proteins.**

**
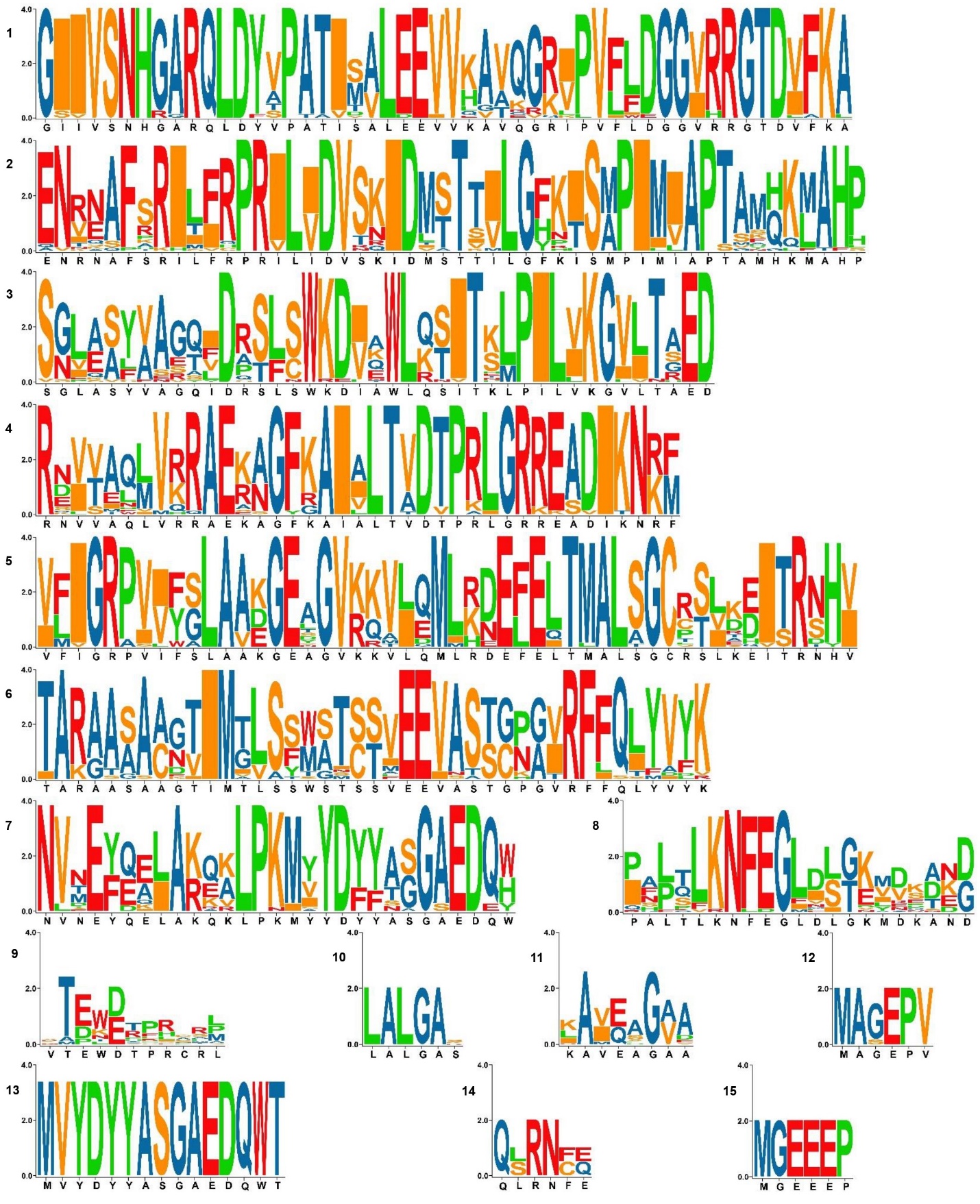
**

**Supplementary material Figure S2 The sequence logos of 15 motifs present in glycolate oxidase proteins.**
